# Transcriptome and metabolite profiling reveals that prolonged drought modulates the phenylpropanoid and terpenoid pathway in white grapes (*Vitis vinifera* L.)

**DOI:** 10.1186/s12870-016-0760-1

**Published:** 2016-03-21

**Authors:** Stefania Savoi, Darren C. J. Wong, Panagiotis Arapitsas, Mara Miculan, Barbara Bucchetti, Enrico Peterlunger, Aaron Fait, Fulvio Mattivi, Simone D. Castellarin

**Affiliations:** Department of Food Quality and Nutrition, Research and Innovation Centre, Fondazione Edmund Mach, Via E. Mach 1, 38010 San Michele all’Adige, Italy; Dipartimento di Scienze Agro-alimentari, Ambientali e Animali, University of Udine, Via delle Scienze 208, 33100 Udine, Italy; Wine Research Centre, The University of British Columbia, 2205 East Mall, Vancouver, BC V6T 1Z4 Canada; Istituto di Genomica Applicata, Parco Scientifco e Tecnologico Luigi Danieli, via Jacopo Linussio 51, 33100 Udine, Italy; The Jacob Blaustein Institutes for Desert Research, Ben-Gurion University of the Negev, Midreshet Ben-Gurion, Israel

**Keywords:** Abiotic stress, Grapevine, Network analysis, RNA sequencing, Transcriptomics, Water deficit

## Abstract

**Background:**

Secondary metabolism contributes to the adaptation of a plant to its environment. In wine grapes, fruit secondary metabolism largely determines wine quality. Climate change is predicted to exacerbate drought events in several viticultural areas, potentially affecting the wine quality. In red grapes, water deficit modulates flavonoid accumulation, leading to major quantitative and compositional changes in the profile of the anthocyanin pigments; in white grapes, the effect of water deficit on secondary metabolism is still largely unknown.

**Results:**

In this study we investigated the impact of water deficit on the secondary metabolism of white grapes using a large scale metabolite and transcript profiling approach in a season characterized by prolonged drought. Irrigated grapevines were compared to non-irrigated grapevines that suffered from water deficit from early stages of berry development to harvest. A large effect of water deficit on fruit secondary metabolism was observed. Increased concentrations of phenylpropanoids, monoterpenes, and tocopherols were detected, while carotenoid and flavonoid accumulations were differentially modulated by water deficit according to the berry developmental stage. The RNA-sequencing analysis carried out on berries collected at three developmental stages—before, at the onset, and at late ripening—indicated that water deficit affected the expression of 4,889 genes. The Gene Ontology category *secondary metabolic process* was overrepresented within up-regulated genes at all the stages of fruit development considered, and within down-regulated genes before ripening. Eighteen phenylpropanoid, 16 flavonoid, 9 carotenoid, and 16 terpenoid structural genes were modulated by water deficit, indicating the transcriptional regulation of these metabolic pathways in fruit exposed to water deficit. An integrated network and promoter analyses identified a transcriptional regulatory module that encompasses terpenoid genes, transcription factors, and enriched drought-responsive elements in the promoter regions of those genes as part of the grapes response to drought.

**Conclusion:**

Our study reveals that grapevine berries respond to drought by modulating several secondary metabolic pathways, and particularly, by stimulating the production of phenylpropanoids, the carotenoid zeaxanthin, and of volatile organic compounds such as monoterpenes, with potential effects on grape and wine antioxidant potential, composition, and sensory features.

**Electronic supplementary material:**

The online version of this article (doi:10.1186/s12870-016-0760-1) contains supplementary material, which is available to authorized users.

## Background

Plant secondary metabolites include more than 200,000 compounds that display a large chemical diversity while accumulating in specific organs, tissues, and cells [1]. They ensure a plant’s survival in the environment by performing a multitude of functions, such as defending plant tissues from pathogens or herbivorous attacks, and aiding reproduction by attracting pollinators or seed dispersers [2]. Berry fruits accumulate a variety of secondary metabolites such as polyphenols, stilbenoids, carotenoids, and free and bound volatile organic compounds (VOCs) [3, 4]. These metabolites affect fruit pigmentation and flavour, and confer to the fruit well-known health benefits. In several fruit crops, the concentration of these metabolites significantly impacts the quality of the fruit and, indeed, the economic value of production. As part of the adaptation mechanism of a plant to its environment, secondary metabolism is sensitive to biotic and abiotic cues [1]. Hence, in agricultural settings the effect of climatic constraints on the accumulation of these metabolites should be taken into consideration for developing cultivation strategies that optimize fruit composition and crop economic value.

Grapes are one of the major fruit crops in the world [5]. Dry and warm Mediterranean climates are considered optimal for wine grape production; in these climates, grapes are often produced without artificial irrigation. However, limited water availability results in reduced vine vigor and fruit growth, significant losses in crop yield, and changes in fruit composition [6]. Moreover, climate change is predicted to exacerbate drought events in several viticultural areas; and Hannah et al. [7] postulate that these phenomena may reduce the viability of viticulture in regions where grapes have been traditionally cultivated.

Grapevine berry secondary metabolism is under strong genetic control and varies among cultivars [8, 9]. Hence, the task of understanding the response of this metabolism to environmental cues is complicated. Several studies have investigated the impact of drought and deficit irrigation strategies on berry secondary metabolism in red grape cultivars, focusing specifically on the accumulation of phenolics. Recently, Hochberg et al. [10] employed large-scale metabolite analyses to investigate the impact of deficit irrigation on this metabolism in Cabernet Sauvignon and Shiraz grapes, and showed cultivar specificity in the magnitude of response. In general, it is recognized that moderate and severe water deficits promote the synthesis and increase the concentration of flavonoids in red grapes, often resulting into better sensory attributes of wines [6]. Besides phenolics, many other secondary metabolites accumulate in the grape berry. These include carotenoids [11] and free and glycosylated VOCs such as C_13_-norisoprenoids, terpenes, aldehydes, ketones, esters, and alcohols [12]. Deluc et al. [13] adopted a microarray platform to investigate differences in the transcriptome response to water deficit between Cabernet Sauvignon, a red grape variety, and Chardonnay, a white grape variety. The study revealed that genes of several secondary metabolic pathways were modulated by water deficit and this metabolic response varied with the cultivar considered. In Chardonnay grapes, water deficit increased the level of expression of one terpene synthase, indicating that terpenes might be part of the metabolic response to water deficit.

The effect of water deficit on secondary metabolism remains largely unexplored in fruits; particularly, very little information is available on the effect of this deficit on the concentration of VOCs, key determinants of fruit economic value, and in the case of wine grapes, of the wine sensory features. Recently, large scale transcript and metabolite analyses have been adopted to reveal the metabolic responses of white grapes to cluster exposure to sunlight and to a biotic stress [14, 15]. In this case study, we employed a large-scale metabolite profiling and RNA-sequencing analyses to evaluate the impact of water deficit on berry secondary metabolism in white grapes in a year characterized by high temperatures and low rainfalls (Additional file 1: Table S1) in a North Italian viticultural region where irrigation is rarely applied to the grapevines. We hypothesize that water deficit may activate the terpenoid pathway and the production of monoterpenes. Two different water regimes were applied to Tocai Friulano vines and the effect of water deficit on the transcriptome program and the phenolic, carotenoid, tocopherol, and free VOC accumulation were investigated at different stages of berry development. Finally, an integrated network analysis was undertaken to investigate the impact of the water deficit on metabolite-metabolite and metabolite-transcript interactions in developing grapes.

## Results

### Impact of irrigation treatments on plant water status, yield, berry growth, berry soluble solids, and titratable acidity

Two irrigation treatments were applied to vines during the season. Irrigated vines (defined as C, controls, henceforward) were weekly irrigated in order to keep their stem water potential (Ψ_Stem_) above −0.8 MPa, whereas vines subjected to deficit irrigation (defined as D, deficit irrigation, henceforward) were not irrigated from fruit set until harvest, unless they displayed signs of extreme water deficit: Ψ_Stem_ lower than −1.5 MPa and fading of the canopy.

**Fig. 1:**
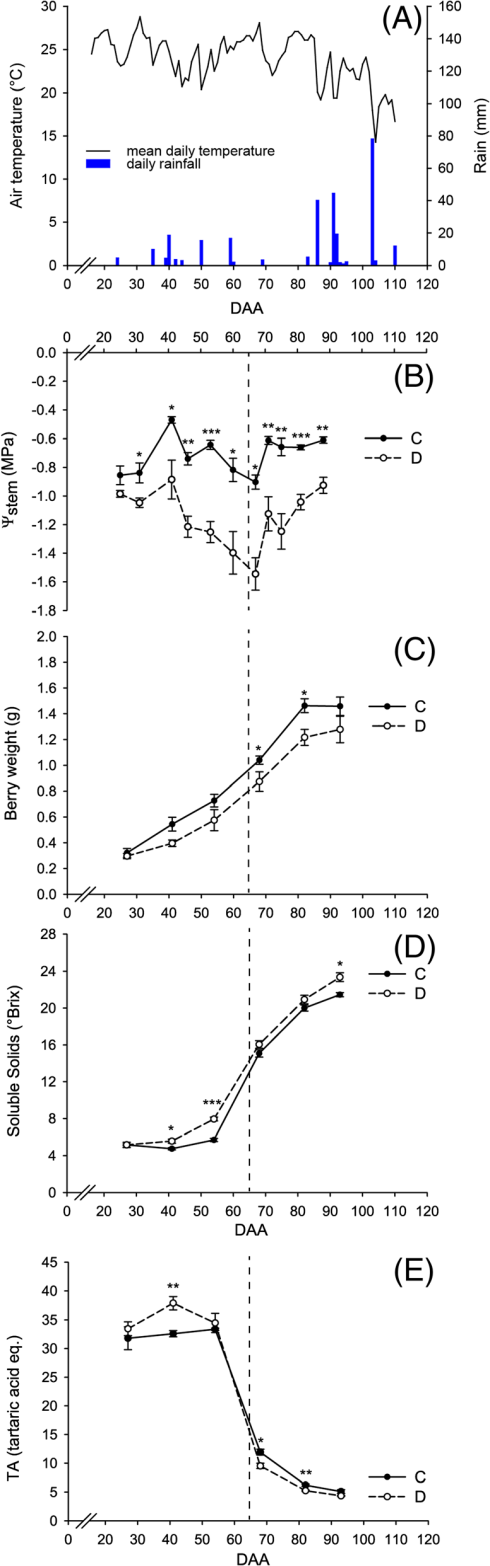
Weather conditions at the experimental site and impact of irrigation treatments on plant and fruit physiology. **a** Daily rainfall and average temperature. Progress of **b** stem water potential (Ψ_Stem_), **c** berry weight, **d** soluble solid accumulation, and **e** titratable acidity in fully irrigated (C) and deficit irrigated (D) vines. *Dotted lines* indicate veraison. *Bars* represent ± SE. *Asterisks* indicate significant differences between treatments at *P* < 0.05 (*), *P* < 0.01 (**), *P* < 0.001 (***) evaluated by one-way ANOVA

Rainfalls during the 2012 season were very limited (Fig. 1a) and mean temperatures peaked just before veraison (the onset of fruit ripening), which was recorded 65 days after anthesis (DAA). Ψ_Stem_ of D vines decreased from early stages of fruit development (Fig. 1b) while Ψ_Stem_ of C vines generally remained above −0.8 MPa. Ψ_Stem_ of D vines reached the seasonal minimum (−1.5 MPa) at 67 DAA. Afterward, three consecutive irrigations together with some rainfalls initiated a partial recovery of Ψ_Stem_ values in D vines.

Irrigation treatments significantly affected vine productivity and D reduced both cluster weight and yield per vine (Additional file 2: Table S2). Moreover, water deficit severely reduced berry weight in D during most part of the season (Fig. 1c), produced increased soluble solids (a good indicator of sugar concentration) before veraison (41 and 54 DAA) and at harvest (93 DAA) (Fig. 1d), and increased and decreased the concentration of acids before (41 DAA) and after (68 and 82 DAA) veraison, respectively, but not at harvest (Fig. 1e).

### Impact of water deficit on secondary metabolites and integrated networks of metabolites

Berries were sampled for secondary metabolite analyses (Additional file 3: Table S3) six times during the season: three times before ripening (27, 41, and 54 DAA), one at the beginning of ripening (68 DAA), one at mid-ripening (82 DAA), and one at late ripening (93 DAA) that coincided with the harvest date of the vineyard. Large scale metabolite analysis identified 27 phenolics, 8 carotenoids, 2 tocopherols, and 37 VOCs. A principal component analysis over the metabolite profiles of the 48 samples analyzed (two treatments x six developmental stages x four biological replicates) was performed (Additional file 4: Figure S1). The analysis indicates that the metabolite profile largely varied based on the berry development, with a sharp distinction between before ripening (27, 41, 54 DAA) and ripening stages (68, 82, 93 DAA), largely driven by the PC1. The irrigation treatment also affected the metabolite profile, with a clear separation of C and D samples at late ripening (93 DAA).

**Fig. 2:**
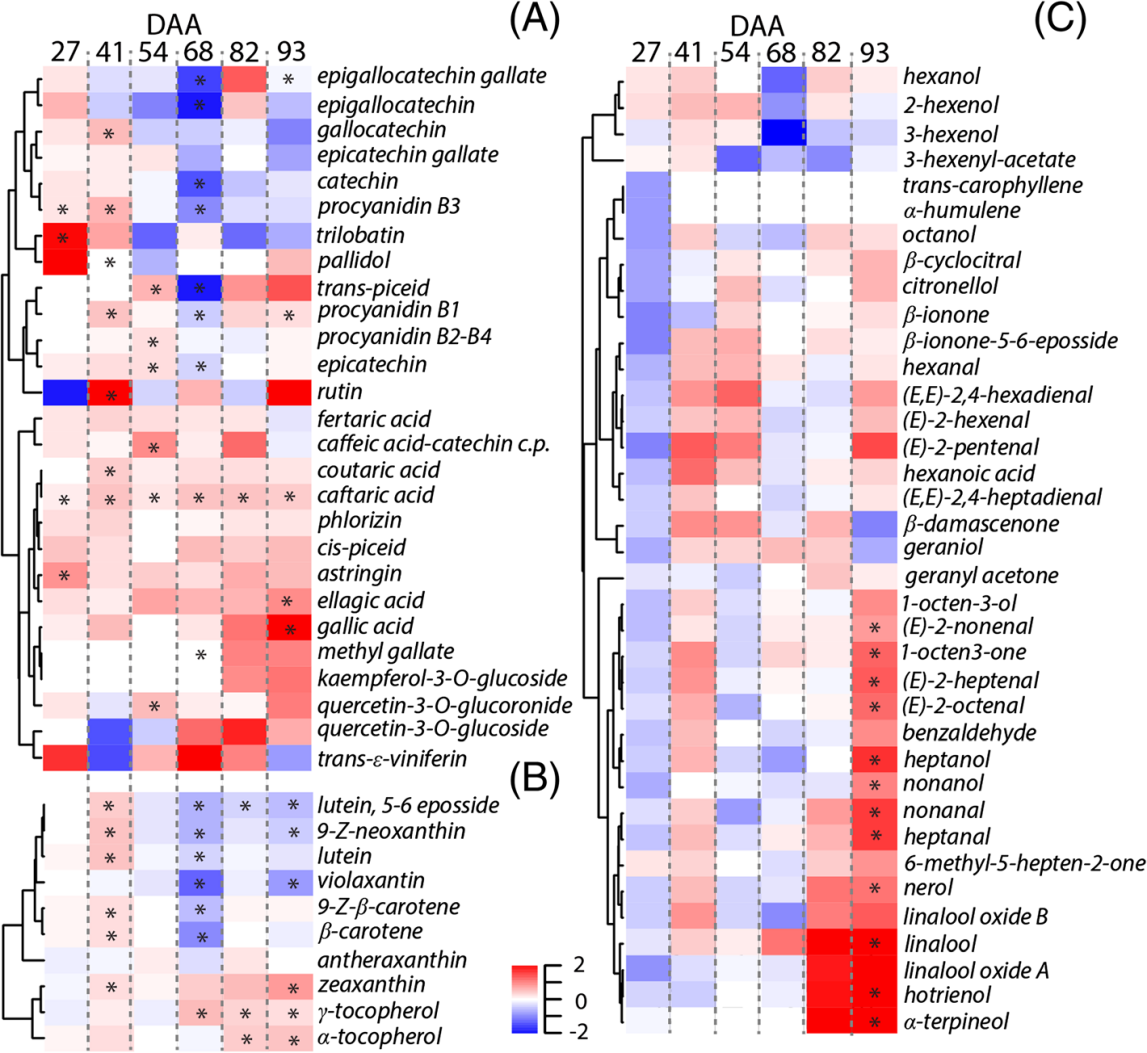
Effect of water deficit on secondary metabolites during fruit development. Heatmaps represent log_2_FC(D/C) of the **a** phenolic, **b** carotenoid and tocopherol, and **c** VOC concentration under water deficit conditions at 27, 41, 54, 68, 82, 93 DAA. *Blue* and *red boxes* indicate lower and higher concentration in D, respectively. *Asterisks* indicate significant differences (*P* < 0.05) between treatments. Metabolites were hierarchically clustered based on their response to water deficit

Water deficit affected the concentration of 20 out of 27 phenolics at one or more developmental stages (Fig. 2a, Additional file 5: Figure S2). Water deficit generally increased the concentration of derivatives of cinnamic and benzoic acids, and modulated the accumulation of flavan-3-ols and proanthocyanidins. Their concentration was increased and decreased by water deficit before (27, 41, and 54 DAA) and after (68, 82, and 93 DAA) veraison, respectively. Limited effects of water deficit on stilbenoid accumulation were observed. In contrast, D largely affected the accumulation of carotenoid and tocopherols in the berry (Fig. 2b, Additional file 6: Figure S3). The concentration of most carotenoids was increased and decreased in D before and after veraison, respectively. Zeaxanthin, α-tocopherol, and γ-tocopherol concentrations were higher in D than in C after veraison. Water deficit also increased the concentration of 12 VOCs (Fig. 2c, Additional file 7: Figure S4) at late ripening (93 DAA). At this stage, D promoted the accumulation of monoterpenes such as hotrienol, linalool, nerol, and α-terpineol.

**Fig. 3:**
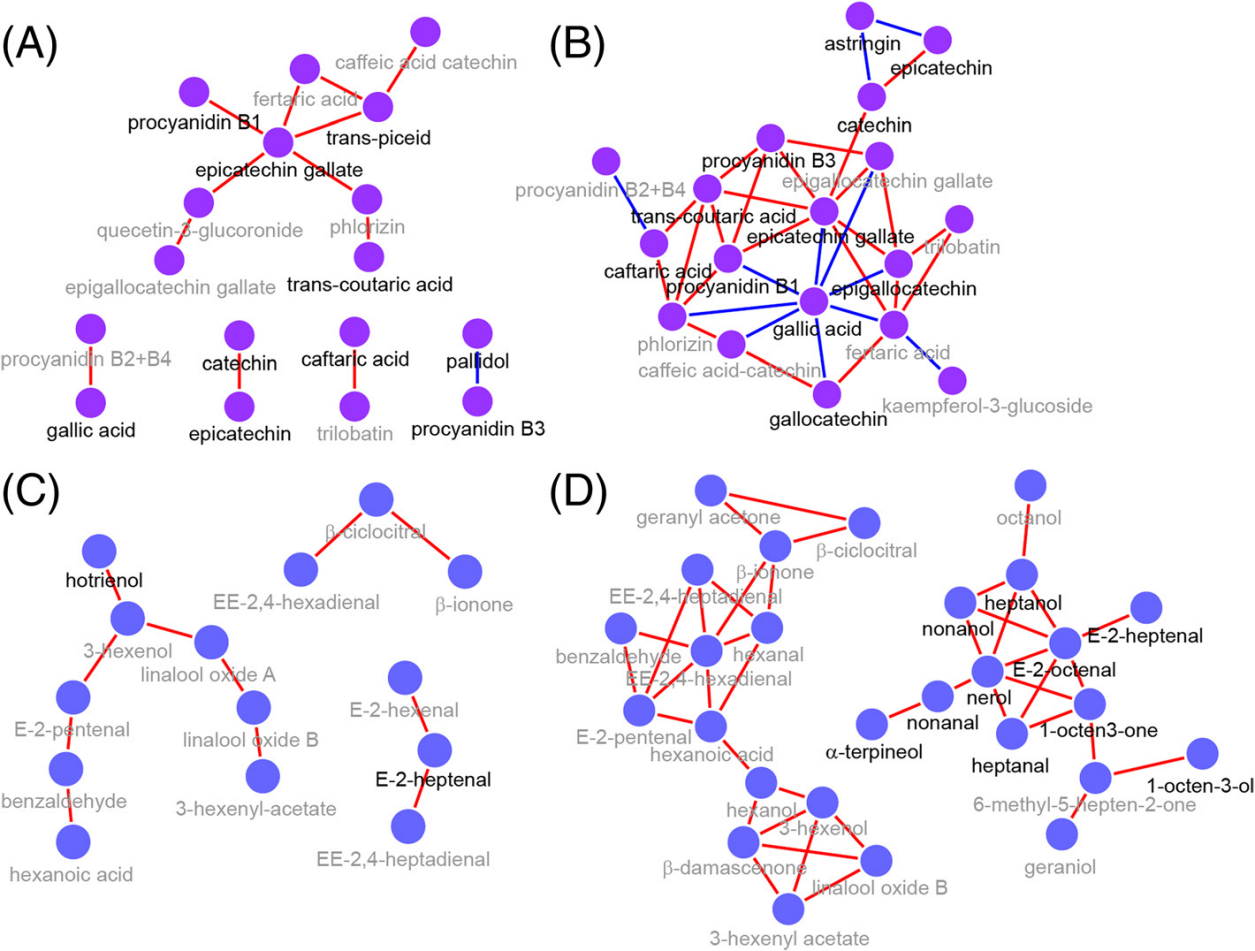
Network representation of phenolics and VOCs in C (**a**, **c**) and D (**b**, **d**) berries during development. Nodes represent ‘metabolites’ and edges represent ‘relationships’ between any two metabolites. Edges colored in ‘*red*’ and ‘*blue*’ represent positive and negative correlations (*P* < 0.001), respectively. Metabolites in *bold* indicate a significant effect of water deficit on the concentration of that metabolite at one or more developmental stages. Number of correlating edges were 13, 35, 11, 42 in (**a**, **b**, **c**, and **d**), respectively. The average node neighborhood was 1.53, 3.89, 1.57, and 3.11 in (**a**, **b**, **c**, and **d**), respectively. The clustering coefficient was 0.08, 0.53, 0.00, and 0.49 in (**a**, **b**, **c**, and **d**), respectively

Differences in metabolic network properties could be observed between C and D (Additional file 8: Table S7A) for the phenolic (Fig. 3a,b) and VOC (Fig. 3c,d) networks, but not for the carotenoid and tocopherol ones (Additional file 9: Figure S5). Water deficit affected the phenolic and VOC network topology by increasing the network connectedness in comparison with the controls. In general, the majority of both C and D metabolite-metabolite correlations are based on positive interactions among nodes, but negative correlations were observed especially under D, in particular for gallic acid. We observed two highly interconnected clusters within the VOC network of D berries; one of these clusters contained many of the VOCs that were significantly modulated under D.

### Impact of water deficit on berry transcriptome

To investigate the molecular changes that take place in the berry under water deficit, and to relate these changes to the observed changes in the berry metabolite profile, we compared the transcriptome of C and D berries at three selected developmental stages, 41 DAA (before ripening), 68 DAA (beginning of ripening), 93 DAA (late ripening).

After filtering for organelles contamination and quality trimming, the average number of unique reads that mapped the V1 version of the grape genome [16] was 25.4 M (Additional file 10: Table S4). Among the 29,971 genes of the grapevine genome, 23,603 (78.8 %) were expressed at 41 DAA, 22,259 (74.4 %) at 68 DAA, and 22,349 (74.7 %) at 93 DAA. At harvest, the number of expressed genes was significantly higher in D (22,655) than in C (22,042).

A strong relationship was found between the RNA-seq and qPCRs gene expression values of 15 genes selected for validating the transcriptomic dataset (Additional file 11:Table S5, Additional file 12: Figure S6). Coefficient of correlation between RNA-seq and qPCR gene expression ranged between 0.792 and 0.999, indicating the reliability of the whole transcriptome assays.

**Fig. 4:**
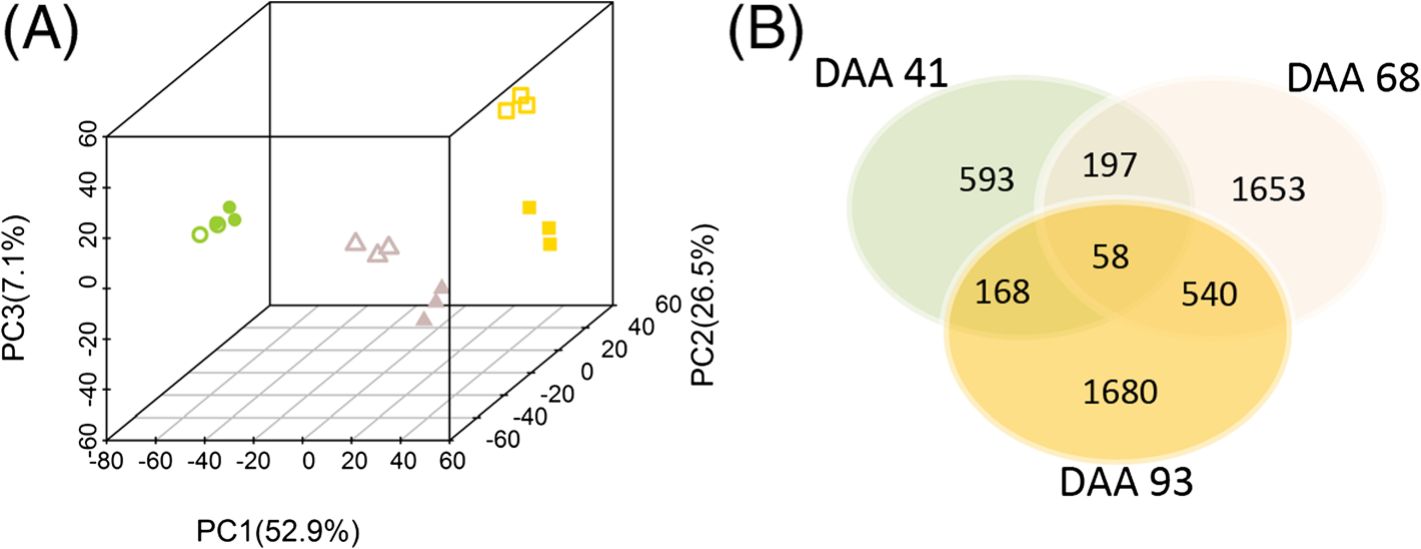
Analysis of the berry transcriptome in fully irrigated (C) and deficit irrigated (D) vines. **a** Principal component analysis (PCA) of the berry transcriptome of 18 independent samples collected from C and D vines at 41, 68, and 93 DAA. Circles, triangles and squares represent berries at 41, 68, and 93 DAA, respectively. Full and open symbols identify C and D berries, respectively. **b** Common and unique DE genes at 41, 68, and 93 DAA are represented in the Venn diagram

A principal component analysis over the transcriptome profiles of the 18 samples analyzed (two treatments x three developmental stages x three biological replicates) was performed (Fig. 4a). The first three principal components explain 52.9, 26.5, and 7.1 % of the variance among samples, respectively. Similarities and differences among berry transcriptomes were mostly driven by the developmental stage when berries were sampled. C and D samples were mixed within the group of the samples harvested at 41 DAA, but were clearly separated at 68 and 93 DAA, with the majority of the variance explained by the second principal component.

The total number of differentially expressed (DE) genes between C and D was 4,889 (Additional file 13: Table S6A, B, C). The number of DE genes changed during fruit development. D modulated the expression of 1,016 genes (316 up-regulated; 700 down-regulated) at 41 DAA, 2,448 genes (1,119 up-regulated; 1,329 down-regulated) at 68 DAA, and 2,446 genes (1,142 up-regulated; 1,304 down-regulated) at 93 DAA. Some genes were differentially regulated in unison among two or three developmental stages (Fig. 4b, Additional file 13: Table S6A, B, C, D).

Seventeen plant GO categories (slim biological processes) were significantly overrepresented among DE genes (Additional file 13: Table S6E). Before ripening (41 DAA), *carbohydrate metabolic process*, *development*, and *response to biotic stress* were the three major Gene Ontology (GO) categories within up-regulated genes, while *response to stress*, *transport*, and *response to abiotic stress* were the major GO categories within down-regulated genes. At the beginning of ripening (68 DAA), *response to stress*, *carbohydrate*, and *response to abiotic stress* were the three major GO categories within up-regulated genes, and *response to stress*, *transport*, and *development* were overrepresented GO categories within down-regulated genes. At late ripening (93 DAA), *response to stress*, *development*, and *response to abiotic stress* were the three major GO categories within up-regulated genes, and *response to stress*, *transport*, and *carbohydrate metabolic process* were enriched GO categories within down-regulated genes. The GO category *secondary metabolic process* was overrepresented within up-regulated genes at all the stages of fruit development considered, and within down-regulated genes at 41 DAA.

### Impact of water deficit on phenylpropanoid, flavonoid, carotenoid, and terpenoid pathway

**Fig. 5:**
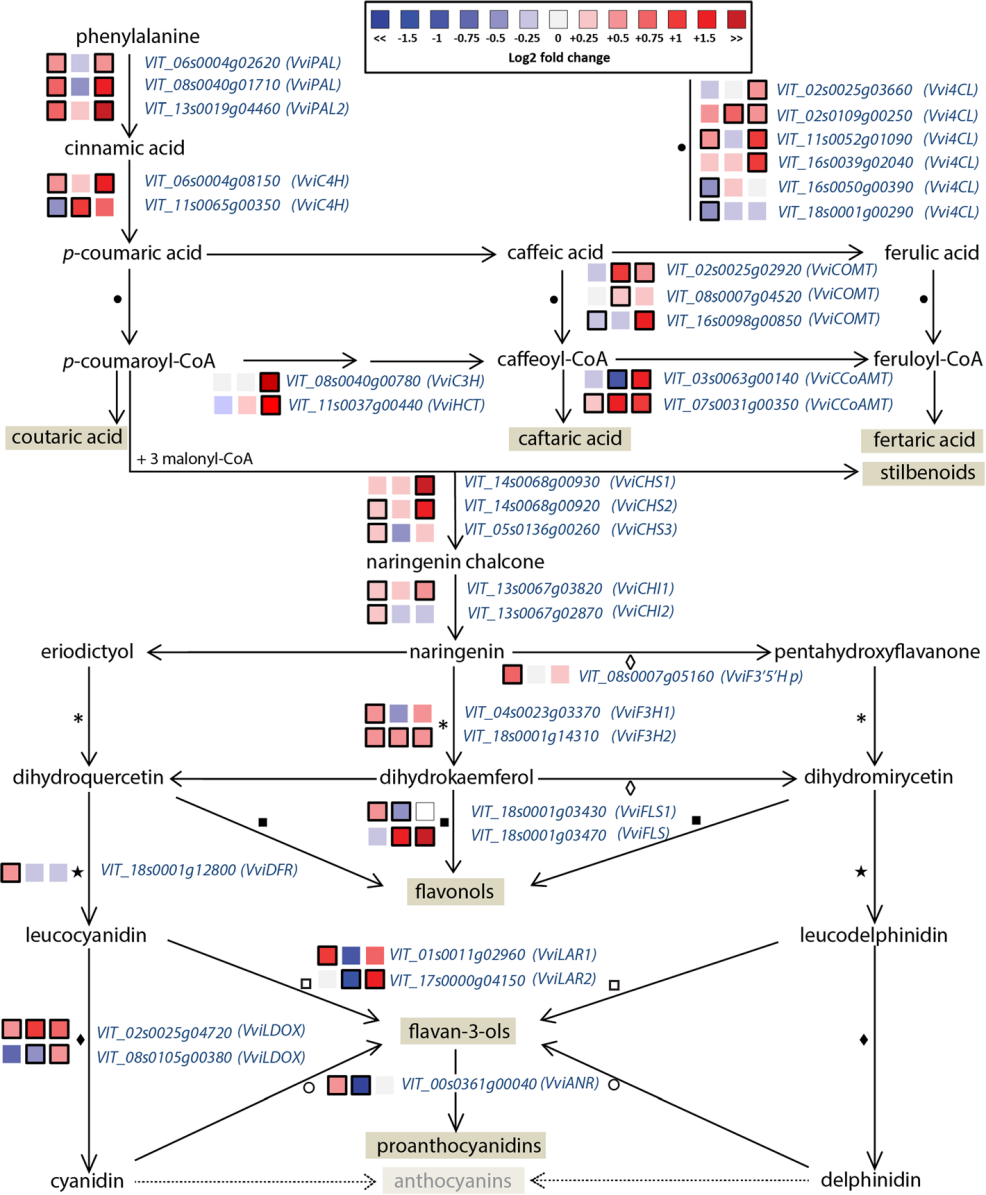
Modulation of phenylpropanoid and flavonoid pathway under water deficit. Log_2_FC (D/C) levels of differential gene expression are presented at 41 (left box), 68 (central box), and 93 (right box) DAA. *Blue* and *red boxes* indicate down- or up-regulation of the gene under water deficit, respectively. *Bold margins* identify significant differences (*P* < 0.05) between treatments. Symbols identify commonly regulated steps of the pathway. Transcript levels, expressed as normalized counts, in C and D berries at 41, 68, and 93 DAA, are reported in Additional file 13: Table S6 F

**Fig. 6:**
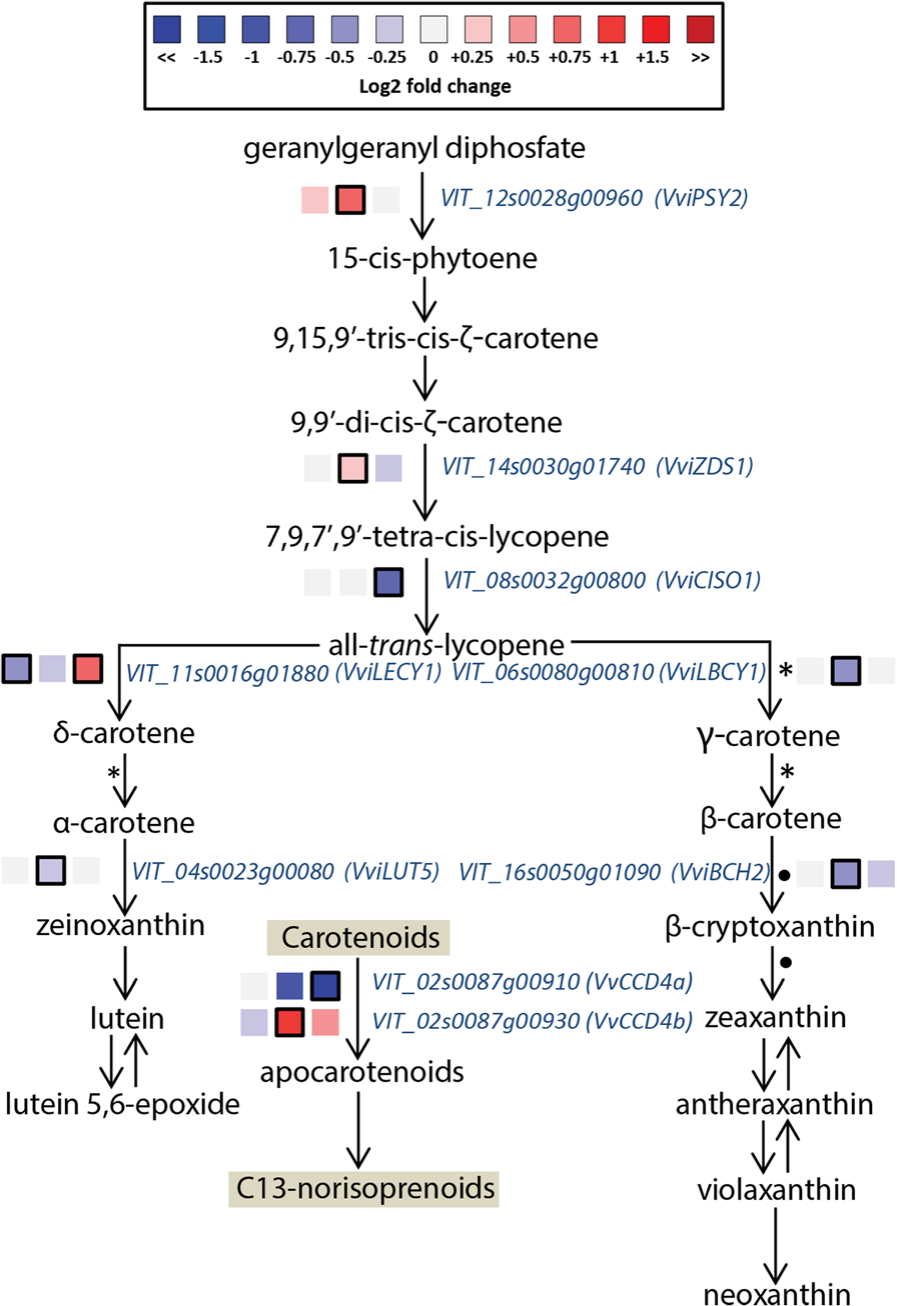
Modulation of carotenoid pathway under water deficit. Log_2_FC (D/C) levels of differential gene expression are presented at 41 (left box), 68 (central box), and 93 (right box) DAA. Blue and red boxes indicate down- or up-regulation of the gene under water deficit, respectively. Bold margins identify significant differences (*P* < 0.05) between treatments. Symbols identify commonly regulated steps of the pathway. Transcript levels, expressed as normalized counts, in C and D berries at 41, 68, and 93 DAA, are reported in Additional file 13: Table S6 G

**Fig. 7:**
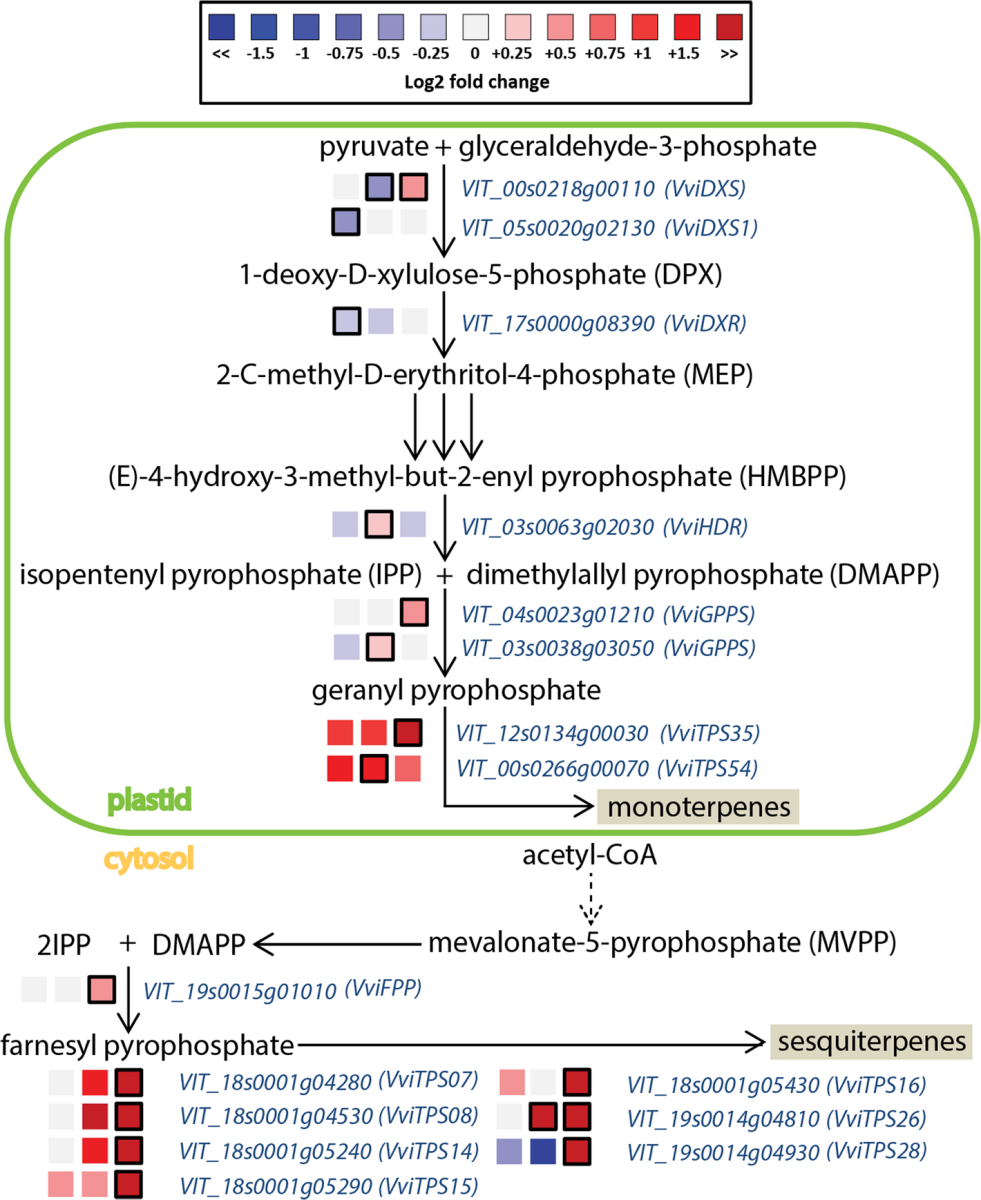
Modulation of terpenoid pathway under water deficit. Log_2_FC (D/C) levels of differential gene expression are presented at 41 (left box), 68 (central box), and 93 (right box) DAA. Blue and red boxes indicate down- or up-regulation of the gene under water deficit, respectively. Bold margins identify significant differences (*P* < 0.05) between treatments. Transcript levels, expressed as normalized counts, in C and D berries at 41, 68, and 93 DAA, are reported in Additional file 13: Table S6 H

Because this study focuses on the impact of water deficit on secondary metabolism, we did identify the DE genes that belonged to the major secondary metabolic pathways in the grapevine berry during development (Additional file 13: Table S6 F, G, H). The impact of water deficit on the expression of these genes was expressed as the log_2_ fold change of the transcript level in D compared to C. Finally, the genes were mapped into the related metabolic pathways (Figs. 5, 6, 7).

Water deficit modulated the expression of many genes that codify for structural enzymes of the phenylpropanoid and flavonoid pathway (Fig. 5). Most of these genes were up-regulated under D, particularly at 41 and 93 DAA.

Among the DE genes, three genes annotated as phenylalanine ammonia lyases (*VviPALs*) were up-regulated by D at 41 and 93 DAA. One *trans*-cinnamate 4-monooxygenase (*VviC4H; VIT_06s0004g08150*) was up-regulated by D at 41 and 93 DAA, while another *VviC4H* (*VIT_11s0065g00350*) was down-regulated at 41 and up-regulated at 68 DAA. Four 4-coumarate-CoA ligase (*Vvi4CL; VIT_02s0025g03660, VIT_02s0109g00250, VIT_11s0052g01090, VIT_16s0039g02040*) were up-regulated by D at different developmental stages. Other two *Vvi4CL* (*VIT_16s0050g00390, VIT_18s0001g00290*) were down-regulated at 41 DAA. One *p*-coumaroyl shikimate 3'-hydroxylase *(VviC3H)* and one hydroxycinnamoyl-CoA:shikimate/quinate hydroxycinnamoyltransferase *(VviHCT)* were up-regulated by D at 93 DAA. Two caffeic acid 3-*O*-metyltransferase (*VviCOMT*) were up-regulated by D: one (*VIT_02s0025g02920*) at 68 and 93 DAA, the other one (*VIT_08s0007g04520*) only at 68 DAA. Finally, a caffeoyl-CoA 3-*O*-methyltransferase (*VviCCoAMT; VIT_03s0063g00140)* was down-regulated at 68 and up-regulated at 93 DAA, while another *VviCCoAMT* (*VIT_07s0031g00350*) was up-regulated at all the three stages of development.

In parallel, water deficit modulated the expression of most structural flavonoid genes; particularly three chalcone synthases (*VviCHSs*), two chalcone isomerases (*VviCHIs*), one flavonoid-3′5′-hydroxylase (*VviF3′5′H*), two flavanone-3-hydroxylases (*VviF3H*s), one dihydroflavonol reductase (*VviDFR*), and two leucoanthocyanidin dioxygenases (*VviLDOX*). All the above genes except one *VviLDOX* (*VIT_08s0105g00380*) were up-regulated by D. The flavonol synthase (VviFLS) is a key enzyme for flavonol production. Water deficit significantly promoted the expression of one *VviFLS* (*VIT_18s0001g03470*) at 68 and 93 DAA while down-regulating the expression of another *VviFLS* (*VIT_18s0001g03430*) at 68 DAA. The leucoanthocyanidin reductase (VviLAR) and anthocyanidin reductase (VviANR) are key regulators of the flavan-3-ol and proanthocyanidin biosynthesis. *VviLAR1* was up-regulated by water deficit at 41 DAA, while *VviLAR2* was down-regulated in the same condition at 68 DAA and up-regulated at 93 DAA. *VviANR* was up-regulated by water deficit at 41 DAA and down-regulated at 68 DAA.

Despite the fact that *VviMyb14* (*VIT_07s0005g03340*) and *VviMyb15* (*VIT_05s0049g01020*)—transcription factors that regulate stilbene synthesis in grapevine [17]—were differentially expressed in D at 68 DAA (Additional file 13: Table S6B), transcript levels of the 48 annotated *VviSTSs* [18] were never affected by water deficit.

The effect of water deficit on the carotenoid pathway was analyzed according to the *Vitis vinifera* carotenoid genes identified by Young et al. [11]. A phytoene synthase gene (*VviPSY2*) was upregulated under water deficit but only at 68 DAA (Fig. 6). The same was observed for a ζ-carotene desaturase (*VviZDS1*). On the contrary, water deficit down-regulated the expression of a lycopene β-cyclase (*VviLBCY*)*,* a β-carotene hydroxylase (*VviBCH2*), and a carotene hydroxylase (*VviLUT5*) at 68 DAA, and of a carotenoid isomerase (*VviCISO1*) at 93 DAA. The expression of a lycopene ε-cyclase (*VviLECY1)* was down-regulated by D at 41 and up-regulated at 93 DAA.

In plants, carotenoids are also the substrate for the production of norisoprenoids. Some C_13_-norisoprenoids, such as β-ionone and β-damascenone, are important determinants of the grape and wine aroma [12]. The enzymes (9,10) (9′,10′) cleavage dioxygenase (CCD4) and (5,6) (5′,6′) (9,10) (9′,10′) cleavage dioxygenase (CCD1) are key enzymes in the norisoprenoid synthesis. In this study, D up-regulated the expression of *VviCCD4b* at 68 DAA and down-regulated the expression of *VviCCD4a* at 93 DAA.

Plant terpenes are synthesized in the plastids through the 2*C*-methyl-D-erythritol-4-phosphate pathway (MEP), and in the cytosol through the mevalonate (MVA) pathway. Water deficit modulated the expression of several genes of the two pathways (Fig. 7). Genes regulating early steps of the MEP pathway, such as one 1-deoxy-D-xylulose-5-phosphate synthase (*VviDXS1*) and the 1-deoxy-D-xylulose-5-phosphate reductoisomerase (*VviDXR*) were down-regulated by D at 41 DAA, while another *VviDXS* was down-regulated at 68 DAA and up-regulated at 93 DAA. Terpene synthases (*VviTPSs*) were generally up-regulated under water deficit, particularly at 93 DAA. The terpene synthases gene family was recently characterized in *Vitis vinifera* [19]. Water deficit modulated the expression of seven terpene synthases of the TPS-a family (*VIT_18s0001g04280*, *VIT_18s0001g04530*, *VIT_18s0001g05240*, *VIT_18s0001g05290*, *VIT_18s0001g05430*, *VIT_19s0014g04810*, *VIT_19s0014g04930*), one of the TPS-b family (*VIT_12s0134g00030*), and one of the TPS-g family (*VIT_00s0266g00070*).

The impact of water deficit on the expression of key genes of the phenylpropanoid, flavonoid, and terpenoid pathway was then investigated at all the six sampling dates with targeted gene expression analyses (Additional file 14: Figure S7). *VviPAL2, VviCHS1, VviFLS*, and *VviANR* were up-regulated by water deficit at several developmental stages in parallel with the observed increase of phenolic concentration under the same conditions (Fig. 2a). Similarly, the expression profile of two *VviTPSs* (*VIT_12s0134g00030 and VIT_19s0014g04930*) indicated that water deficit stimulated a higher synthesis of terpenes from 82 DAA.

### Impact of water deficit on integrated networks of metabolites and transcripts

The increased average node degree, clustering coefficient, and network density between the C and D metabolite-metabolite networks prompted us to perform an association study between metabolites and transcripts in order to reveal the major transcripts that were associated with changes in metabolite networks (Additional file 8: Table S7B, C, D). Emphasis was given on biosynthetic genes of the metabolite pathways considered. The number of positive correlations between phenolic compounds and phenolic biosynthetic genes slightly increased under D particularly because of an increase in the number of correlations within benzoic and cinnamic acid pathway elements (Additional file 8: Table S7C, D). VOC-transcript links were also affected by water deficit. Correlations between geraniol, citronellol, and hotrienol levels and terpenoid transcripts were observed in controls only (Additional file 8: Table S7B, D). In contrast, correlations between nerol and α-terpineol levels and terpenoid transcripts were observed only under water deficit (Additional file 8: Table S7C). Water deficit also modulated the correlations between the non-terpenoid VOCs and the fatty acid related transcripts: reducing them for *(EE)*-2,4-hexadienal and *(E)*-2-pentenal, and increasing them for nonanal, hexanol, and 3-hexenol. The number of carotenoid-transcript correlations was not affected by water deficit.

**Fig. 8:**
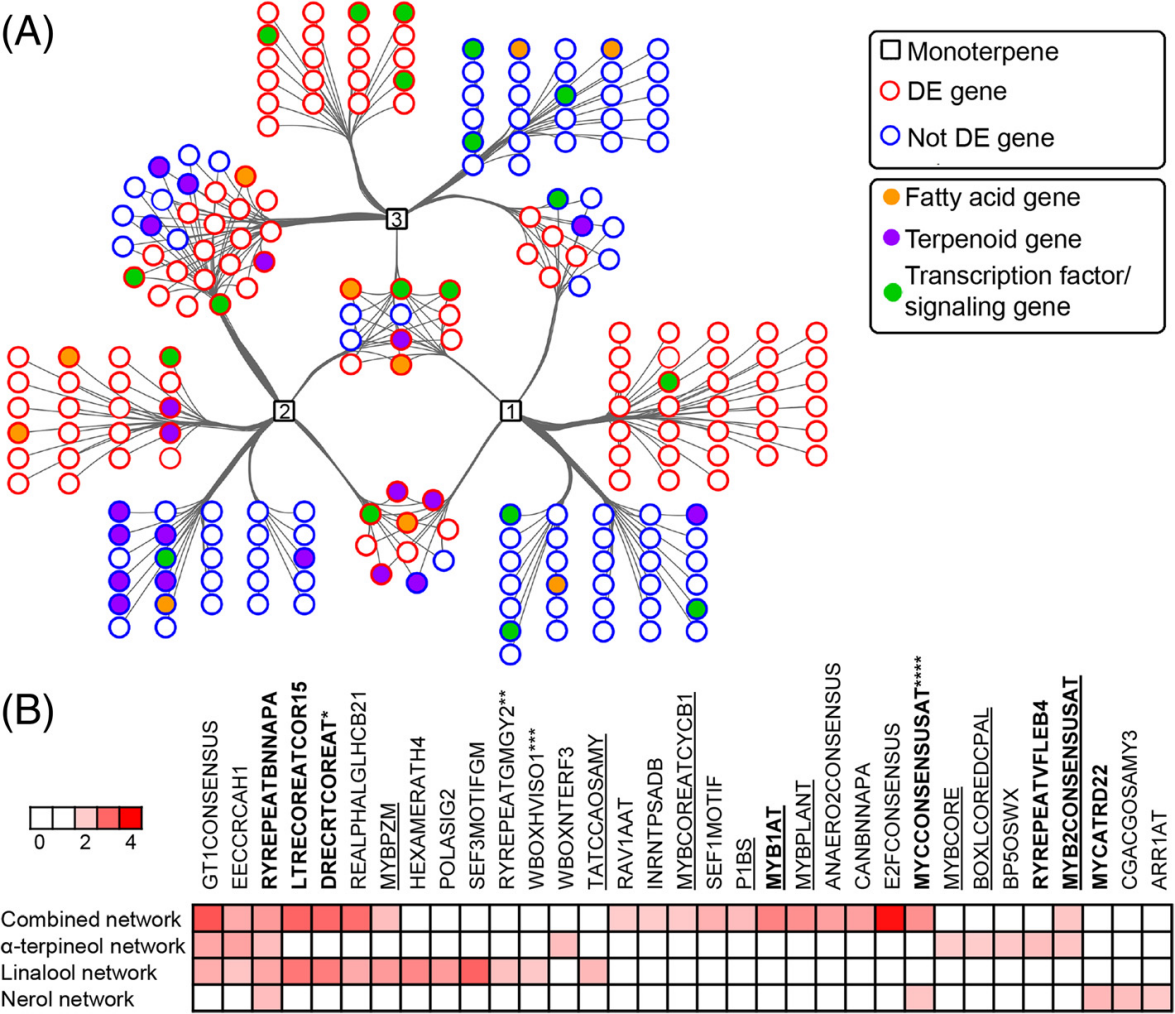
Predicted gene-metabolite networks related to linalool (1), α-terpineol (2), and nerol (3) in grapevine berries during development. **a** Genes and metabolites are represented by circle and square nodes respectively. Edges represent associations (*P* < 0.001) between transcripts and metabolites. The top 100 correlators for each metabolite are shown. Node borders in red represent DE transcripts. Node colors indicate the pathway of the transcripts. **b** Heatmap of *cis*-regulatory elements enriched (*P* < 0.01) within the networks in **a**. *Cis*-regulatory elements in bold and underlined are associated with ABA/drought response and MYB binding, respectively. *Light* and *dark red color* denotes enrichment scores between 2 (*P* < 0.01) and 4 (*P* < 0.0001) respectively. White color represents no significant enrichment. *, **, ***, and **** denotes other PLACE *cis*-regulatory motifs sharing similar consensus sequence with the associated motif (Additional file 8: Table S7F)

The knowledge of the regulation of monoterpene biosynthesis is lacking. Because of the remarkable effect of water deficit on the VOC networks, we furthered our analysis into gene-metabolite relationship focusing on ripening-related monoterpenes induced by water deficit. These included linalool, nerol, and α-terpineol. The gene-metabolite network included the top 100 gene correlators for each of these monoterpenes (Fig. 8a). Among the 222 genes present in the network, 116 genes (52 %) were differentially expressed under water deficit. There were 49, 48, and 64 gene-metabolite relationship that were specific for α-terpineol, nerol, and linalool, respectively. Inspection of the overall network showed that a large proportion of these correlated genes were involved in terpenoid (18), lipid (10), and hormone (7) metabolism, as well as various transport (11) and signaling (13) mechanisms (Additional file 8: Table S7E). Eleven gene-metabolite interactions were found for all the three metabolites and 29 interactions were in common between α-terpineol and nerol. We highlight several potential transcriptional regulators annotated as MYB24 (*VIT_14s0066g01090*), C2H2 Zinc finger (*VIT_07s0005g02190*), and Constans-like 11 (*VIT_19s0014g05120*), which significantly correlated with these monoterpenes. Promoter enrichment analysis of the top 100 correlated transcripts for each metabolite further revealed that many of the genes within each network contain significantly enriched (*P* < 0.01) MYB recognition (such as MYBZM, MYBCOREATCYCB1, MYB1AT, MYBPLANT, MYBCORE, MYB2CONSENSUSAT) and various drought-responsive (RYREPEATBNNAPA, LTRECOREATCOR15, DRECRTCOREAT, MYCCONSENSUSAT, MYCATRD22) motif elements (Fig. 8b, Additional file 8: Table S7F).

## Discussion

The prolonged and severe water deficit imposed in this experiment modulated the accumulation of phenylpropanoids, flavonoids, carotenoids, and several VOCs in the berry.

At present, little information is available on the effect of water deficit on phenolic accumulation in white grapes. Our study indicates that the phenylpropanoid and the flavonoid pathway respond to water deficit at the transcript and metabolite level, and determine a general increase in phenolic concentrations. In red grape cultivars, water deficit strongly promotes accumulation of flavonoids, particularly anthocyanin [13, 20]. Anthocyanin biosynthesis is limited in white grapes; however, these grapes do accumulate other major flavonoids such as flavonols, flavan-3-ols, and proanthocyanidins. Recent studies reported that water deficit increases flavonol concentration [9, 13] and reduces [10] or does not affect proanthocyanidin concentration in grapes [20]. Water deficit can increase the concentration in the berry of secondary metabolites produced in the skin and in the seed by reducing the berry volume and increasing relative skin and seed masses [21–23]. This was not the case in this study, since relative skin and seed masses were not affected by water deficit (Additional file 15: Figure S8). Our gene expression analysis indicated that many phenylpropanoid and flavonoid genes were up-regulated under water deficit, and the modulation of these pathways increased the concentration of derivatives of benzoic and cinnamic acids and of several flavonoids. Interestingly, key structural genes for the flavonol and flavan-3-ol biosynthesis, such as flavonol synthases (*VviFLSs*) and leucoanthocyanidin reductases (*VviLARs*), were up-regulated at late stages of development, while flavonols, flavan-3-ols, and proanthocyanidin increased in concentration under water deficit only at early stages of development (except procyanidin B1, which was also higher at harvest). Similarly, in Cabernet Sauvignon vines exposed to water deficit, *VviLAR*, *VviANR*, and *VviFLS* were up-regulated after the onset of fruit ripening, but no differences in flavonol and proanthocyanidin concentration were observed [20]. Our combined transcript and metabolite data suggest that a competition for precursors between enzymes of the flavonoid and phenylpropanoid pathways is occurring, with phenylpropanoid enzymes being more efficient in directing the substrates into the production of benzoic and cinnamic acid derivatives than the flavonoid enzymes in sequestrating these precursors for the productions of flavonoids, particularly after the onset of fruit ripening when the accumulation of flavan-3-ols and proanthocyanidins decreases dramatically. To this extent, the increase in connectivity (with negative connections) of gallic acid with some flavan-3-ols and proanthocyanidins observed under water deficit highlights the role of this benzoic acid derivative in the drought response, particularly at late stages of development.

Water deficit affected the concentration of carotenoids and tocopherols in the berry, but the modulation of carotenoid genes was much lower than for the phenylpropanoid and flavonoid genes. Carotenoids are normally degraded after the onset of fruit ripening [11], and our data indicate that this degradation is increased under water deficit. However, water deficit increased the concentration of zeaxanthin—the only carotenoid synthesized after the onset of berry ripening (Additional file 6: Figure S3). Zeaxanthin’s role in drought tolerance has been already hypothesized in plants. *Nerium oleander* increased zeaxanthin content in the leaf under water deficit [24], and the enhancement of zeaxanthin levels in the transgenic tobacco lines made plants more tolerant to drought stress [25]. As in our work with berries, previous studies have shown a positive correlation between tocopherol accumulation and water deficit in photosynthesizing tissues [26, 27]. However, we did not observed a consistent upregulation of key genes of the tocopherol pathway under water deficit and, at late stages of ripening (93 DAA), the gene *VviHPT* (*VIT_11s0052g00610*) that encodes for one key enzyme of the pathway was actually down-regulated in D berries (Additional file 13: Table S6), indicating that the accumulation of tocopherols might be affected by post-translational regulation and/or that other unknown genes might be involved in the regulation of this pathway. This result agrees with previous findings in *Arabidopsis*, where wild-type plants subjected to water deficit increased the accumulation of the same tocopherols in the leaves without a parallel modulation of tocopherol biosynthetic genes [27].

The VOC profiling indicated that in Tocai Friulano, VOCs are primarily accumulated at early stages of development (Additional file 7: Figure S4), but prolonged water deficit can stimulate the accumulation of several VOCs at late stages of development. The network analysis revealed that most of the VOCs modulated by water deficit mapped in the same network, but only under water deficit conditions. This suggests that water deficit has a major effect on the accumulation of these VOCs, regardless the pathway they belong to or the type of accumulation pattern they have under normal conditions. Among these VOCs, four monoterpenes—key aromatics of several white grapes [4]—were largely increased under water deficit, in parallel with an up-regulation of key structural genes of the MEP pathway. In particular, key genes for monoterpene production in the grapes such as *VviDXS* and two *VviTPSs* [19, 28] were up-regulated. The induction of monoterpene production under water deficit has been reported in several plants (reviewed in [29]), including the recent studies in grapevine leaves [30, 31], but the information on the effect of drought on monoterpene biosynthesis in fruits (where monoterpenes impact the quality and value of production) is lacking [32]. Besides monoterpene synthases, water deficit also up-regulated seven sesquiterpene synthases [19]. We identified only two sesquiterpenes, α-humulene and trans-caryophyllene, which were accumulated in the berry only at early stages and were not affected by water deficit. However, the molecular data indicate that a more detailed profiling of the sesquiterpene accumulated in the berry is necessary to investigate the role of these compounds in the response to water deficit. Other key odorants of grapes and wines are the carotenoid degradation products C_13_-norisoprenoids that were observed to increase in red grapes subjected to a limiting irrigation regime (reviewed in [12]). Interestingly, despite the higher degradation of carotenoids observed under water deficit, no clear modulation in the concentration of C_13_-norisoprenoids, such as β-damascenone and β-ionone, and of β-cyclocitral—a 7,8 cleavage product of β-carotene [33]—was observed.

Metabolomic studies coupled with network analysis comparing contrasting genotypes, stress perturbations, and tissues have been useful for understanding the mechanism of genotype—environment interactions of plants [34]. Deficit irrigation increased the metabolite network connectivity for primary metabolites in grapevine leaf, but the effect was genotype-dependent for phenolic networks [35]. Network-based analysis conducted here on secondary metabolism revealed that water deficit contributed significantly to restructuring the underlying network properties of fruit metabolites. The higher network connectivity of secondary metabolites observed under water deficit also coincided with the modulation of several genes of the related biosynthetic pathway. It is therefore likely that the observed differential network connectivity between irrigation treatments may be determined by regulation at the transcript level [36, 37]. In support of this hypothesis, we have observed strong gene-metabolite correlations with phenolic and VOC pathway genes. Our results also showed that this observation could be extended to other pathways, such as that for terpenoid biosynthesis. The higher number of positive metabolite-transcript correlations for phenolics and terpenoids further strengthens our finding that those metabolic pathways take part of the grape response to water deficit, producing secondary metabolites that potentially enhance grapevine fitness under this abiotic stress.

In grapes, correlation network analysis has been used recently to ascribe functions to candidate genes potentially involved in the regulation of color development [38, 39]. Ma et al. [40] recently adopted a metabolite-transcript network approach to identify key regulators of the terpenoid pathway in *Artemisia annua*. Similarly, we aimed to identify new genes potentially involved in the regulation of terpenoid metabolism during development and under water deficit. The comprehensive metabolite-transcript network constructed with three key monoterpenes whose synthesis was promoted under water deficit showed strong positive association of these metabolites with terpenoid transcripts. Interestingly, transcripts related to hormone synthesis (salicylic and jasmonic acid) and signaling (auxin and brassinosteroid) were also highly correlated. Terpene levels were significantly increased with the application of BTH (a salicylic acid analog) and methyljasmonate to berries [41] in grapevine. Additionally, our analysis allowed us to identify a transcription factor annotated as *VviMYB24* (*VIT_14s0066g01090*) as a promising regulatory candidate for monoterpene and fatty acid biosynthetic pathways in grapevine. Closer inspection of the annotated *MYB* gene showed high homology towards Arabidopsis *MYB24*, *MYB21*, and *MYB57*, all of which are involved in regulating terpenoid biosynthesis [42]. Recently *MYB24* was found to be strongly up-regulated under solar UV radiation in grape skins, in parallel with the up-regulation of three terpenoid structural genes [43], suggesting a major role in the grapevine berry response to abiotic factors.

Previous studies have shown that terpenoid metabolism responds to light and UV stimuli in berry and leaf tissues [43–45], and we discovered that light-responsive motif elements were significantly enriched throughout the monoterpene gene network (Additional file 8: Table S7F). This data also indicates that the effects of water limitation on berry terpenes may be indirect, in part owing to changes in the fruit microclimate due to a reduction in canopy density. Water deficit can reduce canopy growth determining a higher level of cluster exposure and, by consequence, higher berry temperature [46, 47]. The accumulation of grape volatiles can be influenced by light exposure and temperature. Indeed, exposure of berries to sunlight favors accumulation of norisoprenoids and monoterpenes, and other free VOCs [15, 45, 48–50]. However, recent studies showed that water deficit and ABA treatments significantly increased the monoterpene and sesquiterpene concentration in grapevine leaves [30, 31] even in the absence of UV radiation [30]. Moreover, the enrichment of drought associated elements (e.g., MYB and DRE motifs) in the promoter region of many up-regulated terpenoid genes observed in our study suggests a major direct modulation of the terpenoid pathway at the transcriptional level, possibly via an ABA mediated stimulus. These elements were frequently associated with abiotic stress responses and particularly to drought and ABA regulation [51]. Nevertheless, further tests under more climate-controlled conditions are necessary to reveal to what extent the impact of water deficit on fruit metabolism is due to the modification of berry microclimate.

The stage when deficit is applied and the severity of deficit certainly impact the response of fruit metabolism [32]. In grapes it is known that these factors strongly affect the physiological and metabolic response of the berry to water deficit [6]. In this case study, large effects on fruit metabolism were observed with drought occurring from early stages of berry development to harvest; a condition that also determined a lower yield and higher berry sugar concentration. However, further investigations that compare water deficit imposed at different timings and at different level of severity should be carried out to fully understand the fruit response to this abiotic stress in white grapes, and how this response is consistent among seasons.

## Conclusion

Our study sheds new light into the metabolic mechanisms of fruit response to drought events. Recently it was hypothesized that an overproduction of key odorants, such as terpenoids and the carotenoids-derived norisoprenoids, might be part of the adaptation of white grapes to environmental stresses [5]. Our transcriptome and metabolite analyses showed that, beside the flavonoid pathway, phenylpropanoid and terpenoid pathways can take part in the berry’s response to water deficit in non-pigmented berries; suggesting that an over-production of monoterpenes is part of the fruit response to drought. Our network and promoter analyses highlighted a transcriptional regulatory mechanism that encompasses terpenoid genes, transcription factors, and drought-responsive elements enriched in the promoter regions of those genes; this mechanism might be the basis of monoterpenes overproduction. Overall, these results indicate that water deficit conditions can potentially impact the quality of white wines by increasing the accumulation of potential antioxidant and flavor compounds (e.g., derivatives of benzoic and cinnamic acids, zeaxanthin, and monoterpenes) in the grapes. These results are also pivotal to future studies that evaluate the impact of deficit irrigation strategies on wine quality.

## Methods

### Field experiment, physiological measurements, and sample preparation

The field experiment was conducted in 2012 in a vineyard at the University of Udine’s (Italy) experimental farm (46°01'52.3"N 13°13'30.6"E). The field experiment was conducted in accordance with local legislation and no specific permission was required for the study.

Climatological data were recorded during the experiment by an automated weather station located 100 m from the experimental site. Monthly mean temperatures and amount of rainfalls measured in 2012, as well as the averages of the 2000–2012 period, are shown in Additional file 1: Table S1. Seven years old Tocai Friulano (also known as Sauvignon vert and Sauvignonasse in Chile and France, respectively) grapevines grafted onto SO4 were planted at 2.5 m x 1.0 m spacing in north–south oriented rows, and trained to a cane-pruned ‘Guyot’ system. Two irrigation treatments were established. Control (C) vines were irrigated in order to maintain midday stem water potential (ψ_Stem_) above −0.8 MPa. Deficit irrigated (D) vines were not irrigated unless the ψ_Stem_ was measured lower than −1.5 MPa. Plant water status was monitored weekly by measuring ψ_Stem_ using a Scholander pressure chamber [52]. Irrigation was supplied when rainfall in the preceding week was below 100 % ETc. or ψ_Stem_ was measured lower than −1.5 MPa, as discussed above. A surface drip irrigation system with emitters (0.5 m x 2.5 m) set to an 8 L h^−1^ application rate was used. At the maximum rate, water was supplied at approximately 40 L per vine per week. Due to a prolonged drought, irrigation (20 L per vine) was applied to D vines at 67, 70, 76 days after anthesis (DAA) in order to mitigate the extreme water deficit. Each irrigation treatment was replicated on four plots of six vines each, arranged in a completely randomized design. No effect of the irrigation treatments was observed on the number of shoots and clusters per vine.

Samplings were carried out at 27, 41, 54, 68, 82, and 93 DAA. Three sets of berries were randomly collected from each plot. The first set of 60 berries was used to measure berry weight, total soluble solid concentration, and titratable acidity. The second one of 10 berries was used to measure skin and seed weights and calculate the relative skin and seed masses. The third set of 40 berries was used for transcript and metabolite analyses. Berries were carefully trimmed off the cluster at the pedicel with a pair of scissors, quickly brought to the laboratory, weighed, and processed for soluble solids and titratable acidity [52] or immediately frozen at −80 °C for transcript and metabolite analyses. Before metabolite and RNA extractions, pedicel was removed with a scalpel and berries were ground to a fine powder under liquid nitrogen using an analytical mill (IKA®-Werke GMbH & CO). One quality control (QC) sample was prepared by pooling an aliquot of all the samples and was used for QC runs in the metabolite analyses.

Grapes were harvested for commercial wine production at 93 DAA, when titratable acidity reached approximately 5 mg/L in both treatments; yield per vine, number of clusters per vine, and cluster weight were recorded.

### Metabolite analyses

Phenolic compounds were determined accordingly to Vrhovsek et al. [53] with some modifications. Briefly, 0.8 mL of chloroform and 1.2 mL of a mix of methanol and water (2:1) were added to one gram of frozen powder of ground berries. A 50 μL aliquot of *o*-coumaric acid solution (2 mg/mL in MeOH) was added as an internal standard. The extraction mixture was shaken for 15 min on an orbital shaker (Grant-Bio Rotator PTR-60) and then centrifuged for 10 min at 1000 *g*. The upper aqueous-methanolic phase was collected. The extraction was repeated by adding 1.2 mL of methanol and water. The aqueous-methanolic phase was collected and combined with the previous one, brought to a final volume of 5 mL with Milli-Q water, and filtered with a 0.2 μm PTFE filter (Millipore). The chromatographic analysis was carried out using a Waters Acquity UPLC system (Milford) coupled to a Waters Xevo triple-quadrupole mass spectrometer detector (Milford). Compounds were identified based on their reference standard, retention time, and qualifier and quantifier ion, and were quantified by their calibration curve.

Carotenoids and tocopherols were analyzed accordingly to Wehrens et al. [54]. Briefly, the chloroform phase of the extraction solution described above was collected. Twenty μL of *trans*-β-apo-8′-carotenal (25 μg/mL) was used as internal standard. Ten μL of a 0.1 % triethylamine solution was added to prevent rearrangement of carotenoids. After extraction, samples were dried with N_2_, and stored at −80 °C until analysis. Dried samples were suspended in 50 μL of ethyl acetate, and transferred to dark vials. The chromatographic analysis was performed in a 1290 Infinity Binary UPLC (Agilent) equipped with a RP C30 3 μm column coupled to a 20 x 4.6 mm C30 guard column. Spectra components and elution profiles were determined as in Wehrens et al. [54]. Compounds were quantified from linear calibration curves built with standard solutions.

Free (non-glycosylated) VOCs were analyzed accordingly to Fedrizzi et al. [55] with some modifications. On the day of analysis, four grams of frozen grape powder were weighed out in a 20 mL SPME dark-glass vial. Three grams of NaCl, 15 mg of citric acid, 15 mg of ascorbic acid, 50 μL of sodium azide, and 7 mL of milliQ water were added to the sample. Fifty μL of a solution containing five internal standards, d_10_-4-methyl-3-penten-2-one (1 g/L), d_11_-ethyl hexanoate (1 g/L), d_16_-octanal (1 g/L), d_8_-acetophenone (1 g/L), d_7_-benzyl alcohol (1 g/L), was added to each sample. Prior to injection, the sample was pre-incubated at 60 °C in a SMM Single Incubator (Chromtech) for 10 min stirring at 450 rpm. Next, the sample was incubated in the same conditions for 40 min with a DVB-CAR-PDMS 50/30 μm x 2 cm (Supelco) fiber in the headspace for absorption. Free VOCs were thermally desorbed in splitless mode for 4 min at 250 °C. Extractions and injections were carried out with a CTC Combi-PAL autosampler (Zwingen). The analysis was performed with a Trace GC Ultra gas chromatograph (Thermo Scientific) coupled to a TSQ Quantum Tandem mass spectrometer. GC separation was performed on a 30 m Stabilwax (Restek Corporation) capillary column with an internal diameter of 0.25 mm and a film thickness of 0.25 μm with the conditions described in Fedrizzi et al. [55]. VOCs were identified by comparing the retention times of individual peaks with the retention times of their reference standards, and by identifying the mass spectra using the NIST library. The ratio of each VOC area to the d_16_-octanal internal standard area was considered to reduce technical variability among extractions and chromatographic runs and VOCs quantity were expressed as μg/kg of berry of d_16_-octanal equivalents.

Extractions and injections of the samples were performed in a random sequence and QC samples were injected at the beginning of the sequence and every six sample injections.

A list of the secondary metabolites analyzed in this study is reported in Additional file 3: Table S3.

### RNA extraction and RNA sequencing analysis

Samples collected at 41, 68, and 93 DAA were selected for transcriptome analyses. Three biological replicates per treatment were considered. Total RNA was extracted with the ‘Spectrum Plant total RNA’ kit (Sigma-Aldrich) from 0.2 g of ground berries. The quantity and quality of the RNA were determined with a Caliper LabChip® GX (Perkin-Elmer).

Library preparation was performed using the TruSeq RNA Sample Prep Kit v2.0 according to the manufacturer’s instructions (Illumina). Libraries were quantified using a 2100 Bioanalyzer (Agilent Technologies). Multiplexed cDNA libraries were pooled in equimolar amounts, and clonal clusters were generated using Cbot (Illumina). Sequencing was performed with an Illumina HiSeq 2000 platform (Illumina pipeline 1.8.2) at IGA Technology Services (Udine, Italy).

An average of 28.9 M 50-nt single-end reads was generated per sample (Additional file 10: Table S4). Trimming for quality and length, and filtering for mitochondria and chloroplast contamination were performed by the ERNE package version 1.2 tool ERNE-FILTER [56]. The minimum PHRED score accepted for trimming was 20, and reads shorter than 40 bp were discarded. Reads were aligned against the reference grapevine genome PN40024 12x [16] using the software TopHat version 2.0.6 [57] with default parameters. Aligned reads were counted with a htseq-count (version 0.6.0) in intersection-non-empty mode for overlap resolution [58]. *Vitis vinifera* annotation GTF-file (V1) was downloaded from Ensembl Plants website. Differentially expressed (DE) genes (false discovery rate less than 0.05) analysis was performed with the R package DeSeq2 [59]. Functional annotations of genes were retrieved from Grimplet et al. [60] and VitisCyc [61]. Gene ontology analyses were carried out for each sampling. Overrepresented genes categories were identified with the BINGO app 3.0.2 of Cytoscape 3.1.1 [62]. PlantGoSlim categories, referred to biological processes, were used to run the gene enrichment analysis using a hypergeometric test with a significance threshold of 0.05 after Benjamini and Hochberg false discovery rate correction.

### Quantitative real-time polymerase chain reaction

The validation of RNA-Seq data was carried out on a set of DE genes using the quantitative real-time polymerase chain reaction (qPCR) technique. RNA was extracted from independent biological samples collected at the same stage than the ones used for RNA-Seq analysis. The reverse transcription of RNA samples was performed with the QuantiTect Reverse Transcription Kit (Qiagen) and the Quantiscript Reverse Transcriptase (Qiagen). Specific primers for 15 selected genes were designed with Primer3web version 4.0.0 (Additional file 11: Table S5). qPCR reactions, conditions, and calculation of relative expression values were carried out as in Falginella et al. [63]. The annealing temperature was 58 °C for all primer pairs except the *VviUbiquitin* housekeeping gene pair, which annealed at 56 °C. Correlation analysis based on the Pearson Correlation Coefficient (PCC) was carried out between the RNA-seq normalized counts and qPCR relative gene expression (Additional file 11: Table S5, Additional file 12: Figure S6).

qPCR was also carried out to determine the level of expression of selected structural genes of the phenylpropanoid, flavonoid, and terpenoid pathway at each sampling date.

### Statistical, network, and promoter analyses

A one-way ANOVA was performed using JMP 7 (SAS Institute Inc.) to detect significant differences (*P* < 0.05) between irrigation treatments at each sampling point. Heatmaps representing log_2_ fold change (log_2_FC) of metabolite concentrations between treatments (D/C) and principal component analysis (PCA) on the metabolite profiles and on the entire transcriptome dataset were constructed and performed, respectively, using R software.

The metabolite correlation network was constructed for each condition (C and D) using all 74 metabolite accumulation profiles separately. The PCC was used as an index of similarity between any two variables (i.e. metabolites). Correlation pairs were deemed statistically significant when the |PCC| > 0.8 and *P* value < 0.001 (2,000 permutations). The Cytoscape software (version 3.1.1) [64] was used for network visualization and analysis of network properties such as the average node degree, clustering coefficient, and network density. Additionally, the two matrices (C and D) of metabolite and transcript datasets were merged and used for the construction of a global metabolite-transcript network focused on structural genes of phenylpropanoid, flavonoid, carotenoid, fatty acid, and terpenoid pathway. Selected networks were constructed for three monoterpenes (linalool, nerol, and α-terpineol) considering the top 100 correlating genes. All calculations and permutation tests were performed in R using the ‘rsgcc’ package [65].

Promoter motif enrichment analysis was conducted as described previously in Ma et al. [66]. A total of 29,839 grapevine promoter sequences (1 kb upstream of the 5′ UTR) based on the 12x grapevine genome assembly were retrieved from Gramene v45 (http://www.gramene.org/). Known *cis*-regulatory motifs of plants were retrieved from PLACE [67]. Enrichment of motifs was validated using the hypergeometric distribution test. *Cis*-regulatory motifs were considered significantly enriched if the associated *P* value was < 0.01 and at least 10 promoters were associated with the given motif.

## Availability of Data and Materials

All raw sequence reads have been deposited in NCBI Sequence Read Archive (http://www.ncbi.nlm.nih.gov/sra). The BioProject and SRA accession are PRJNA313234 and SRP070855, respectively.

## Additional files

Additional file 1: Table S1. Comparison of the climatological data in the season of the study (2012) with data of the 2000–2012 period. (DOC 35 kb) Additional file 2: Table S2. Effect of water deficit on crop production. Yield per vine, clusters per vine, and cluster weight of fully irrigated (C, controls) and deficit irrigated (D, water deficit) grapevines reported as the average ± the standard error. Numbers in bold indicate significant differences between treatments (*P* < 0.05) identified by one-way ANOVA (*n* = 4). (DOC 29 kb) Additional file 3: Table S3. List of phenolics, carotenoids, tocopherols and free VOCs identified in the study. (DOC 88 kb) Additional file 4: Figure S1. Principal component analysis (PCA) of the berry secondary metabolite profile of 48 independent samples collected from C and D vines at 27, 41, 54, 68, 82, and 93 DAA. Full and open symbols identify C and D berries, respectively. (PNG 27 kb) Additional file 5: Figure S2. Phenolic profiling. Trends of phenolic concentrations in C and D berries during fruit development. (DOCX 358 kb) Additional file 6: Figure S3. Carotenoid and tocopherol profiling. Trends of carotenoid and tocopherol concentrations in C and D berries during fruit development. (DOCX 163 kb) Additional file 7: Figure S4. VOC profiling. Trends of VOC concentrations in C and D berries during fruit development. (DOCX 468 kb) Additional file 8: Table S7. Network analysis metrics. Detailed information concerning the various metabolite/transcript annotations, correlation relationships (PCC), and statistical significance (empirical *P* value) are depicted for the global (C and D) metabolite-metabolite network (A) and metabolite-transcript network in C (B) and D (C). Frequency tables for the significant metabolite-transcript correlations from (B) and (C) are summarized in (D). Correlation values (PCC, statistical significance of PCC, and correlation ranks) for the predicted monoterpene (linalool, nerol, and α-terpineol)-transcript network reported in Fig. 8a (E). Contingency tables containing enriched (*P* < 0.01) PLACE *cis*-regulatory elements and associated information (e.g., motif description, occurrence of motif within promoter regions) identified in the combined monoterpene-transcript network presented in Fig. 8a (F). (XLSX 91 kb) Additional file 9: Figure S5. Carotenoid network. Network representation of carotenoids in C (A) and D (B) berries during development. Nodes represent ‘metabolites’ and edges represent ‘relationships’ between any two metabolites. Edges colored in red and blue represent significant (*P* < 0.001) positive and negative correlations, respectively. Metabolites in bold indicate a significant effect (*P* < 0.05) of water deficit on the concentration of that metabolite at one or more developmental stages. Number of correlating edges, average node neighborhood, and clustering coefficient were similar between C and D networks. (PNG 63 kb) Additional file 10: Table S4. RNA sequencing analysis metrics. Transcriptome analyses were performed in C and D berries at three selected berry developmental stages (41 DAA, before ripening; 68 DAA, beginning of ripening; 93 DAA, late ripening) using an Illumina HiSeq platform. (DOC 54 kb) Additional file 11: Table S5. List of genes assayed for expression by qPCR. For each, the coefficient of correlation between RNA-Seq data and qPCR data, the *P* value, forward and reverse primers used, and literature references are shown. (DOC 38 kb) Additional file 12: Figure S6. Scatterplot of the correlation between the fold changes (log_2_(D/C)) in the expression levels of the 15 genes reported in Additional file 11: Table S5 obtained by RNA-seq and qPCR analyses. (TIF 1009 kb) Additional file 13: Table S6. Differentially expressed genes. Summary of differentially expressed genes (*P* < 0.05) and associated information (12x V1 identification number, predicted annotation, fold change values, and adjusted *P* value) identified between C and D at 41 (A), 68 (B), and 93 (C) days after anthesis (DAA). The ‘Venn’ column in A, B, C identifies where each gene maps in the Venn diagram of Fig. 4. List of the genes differentially expressed at all the developmental stages (D). Plant gene ontology (GO) slim biological process categories enriched (*P* < 0.05) within significantly up- and down-regulated genes at 41, 68, and 93 DAA are represented as the relative (%) contribution of genes ascribed to that biological process over the total DE genes at each developmental stage (E). Level of expression of DE genes of the phenylpropanoid and flavonoid (F), carotenoid (G), and terpenoid (H) pathways at 41, 68, 93 DAA in C and D berries. Red-blue color scale identifies high (red) and low (blue) levels of expression. (XLSX 1175 kb) Additional file 14: Figure S7. Target gene expression analysis. Impact of water deficit on transcript abundance of selected genes of the phenylpropanoid, flavonoid, and terpenoid pathway. Using qPCR, gene expression was analyzed at each sampling time. Gene expression levels analyzed with RNA-sequencing at 41, 68, and 93 DAA are reported in inset graphs for comparison. Bars represent ± SE. Asterisks indicate significant differences between treatments at *P* < 0.05 (*). (PNG 275 kb) Additional file 15: Figure S8. Evolution of the relative skin and seed masses expressed as % of berry fresh weight (FW) across development in C and D berries. (TIF 1797 kb)

## Abbreviations

4CL: 4-coumarate-CoA ligase
ANR: anthocyanidin reductase
BCH: β-carotene hydroxylase
C: control
C3H: p-coumaroyl shikimate 3'-hydroxylase
C4H: trans-cinnamate 4-monooxygenase
CCD1: (5,6) (5′,6′) (9,10) (9′,10′) cleavage dioxygenase
CCD4: (9,10) (9′,10′) cleavage dioxygenase
CCoAMT: caffeoyl-CoA 3-O-methyltransferase
CHI: chalcone isomerase
CHS: chalcone synthase
CISO: carotenoid isomerase
COMT: caffeic acid 3-O-metyltransferase
D: deficit irrigation
DAA: days after anthesis
DE: differentially expressed
DFR: dihydroflavonol reductase
DVB-CAR-PDMS: divinylbenzene-carboxen-polydimethylsiloxane
DXR: 1-deoxy-D-xylulose-5-phosphate reductoisomerase
DXS: 1-deoxy-D-xylulose-5-phosphate synthase
ETc.: crop evapotranspiration
F3′5′H: flavonoid-3′5′-hydroxylase
F3H: flavanone-3-hydroxylase
FLS: flavonol synthase
FW: fresh weight
GO: gene ontology
HCT: hydroxycinnamoyl-CoA:shikimate/quinate hydroxycinnamoyltransferase
HPLC-DAD: high performance liquid chromatography-diode array detector
HPT: homogentisate phytyl transferase
HS-SPME-GC-MS: headspace-solid phase microextraction-gas chromatography–mass spectrometry
LAR: leucoanthocyanidin reductase
LBCY: lycopene β-cyclase
LDOX: leucoanthocyanidin dioxygenases
LECY: lycopene ε-cyclase
LUT: carotene hydroxylase
MEP: 2C-methyl-D-erythritol-4-phosphate
MVA: mevalonate
PAL: phenylalanine ammonia lyase
PCA: principal component analysis
PCC: Pearson correlation coefficient
PSY: phytoene synthase
QC: quality control
qPCR: quantitative PCR
RNA-seq: RNA sequencing
STS: stilbene synthase
TPS: terpene synthase
UFGT: UDP-glucose:flavonoid 3-O-glucosyltransferase
UHPLC-MS/MS: ultra-high performance liquid chromatography-tandem mass spectrometer
VOCs: volatile organic compounds
Vvi: Vitis vinifera
ZDS: **ζ**-Carotene desaturase
Ψ_Stem_: stem water potential
