# Supplementary material for: Transcriptome and metabolite profiling reveals that prolonged drought modulates the phenylpropanoid and terpenoid pathway in white grapes (Vitis vinifera L.)
Source: BMC Plant Biol. 2016 Mar 21;16:67. doi: 10.1186/s12870-016-0760-1 (PMC4802899; doi:10.1186/s12870-016-0760-1)
Supplement: Additional file 1: Table S1. — Comparison of the climatological data in the season of the study (2012) with data of the 2000–2012 period. (DOC 35 kb) [file 12870_2016_760_MOESM1_ESM.doc]

| **Table S1.** Comparison of the climatological data in the season of the study (2012) with data of the 2000-2012 period. | | | | | | |
| --- | --- | --- | --- | --- | --- | --- |
| **Month** | **Rain**  **2000-2012**  **(mm)** | **Rain**  **2012 (mm)** | **Δ Rain (mm)** | **Mean Temperatures 2000-2012**  **(°C)** | **Mean Temperatures 2012**  **(°C)** | **Δ Mean Temperatures (°C)** |
| May | 114.7 | 87.7 | - 27.0 | 18.2 | 17.6 | - 0.6 |
| June | 111.4 | 114.3 | + 2.9 | 21.8 | 22.3 | + 0.5 |
| July | 122.7 | 72.2 | - 50.5 | 23.3 | 24.4 | + 1.0 |
| August | 141.0 | 94.0 | - 47.0 | 23.1 | 24.8 | + 1.7 |
| September | 149.0 | 201.3 | + 52.3 | 18.5 | 19.7 | + 1.2 |
| Total/Average Δ | 638.7 | 569.5 | - 69.2 | 21.0 | 21.7 | + 0.8 |
