# Supplementary material for: Transcriptome and metabolite profiling reveals that prolonged drought modulates the phenylpropanoid and terpenoid pathway in white grapes (Vitis vinifera L.)
Source: BMC Plant Biol. 2016 Mar 21;16:67. doi: 10.1186/s12870-016-0760-1 (PMC4802899; doi:10.1186/s12870-016-0760-1)
Supplement: Additional file 2: Table S2. — Effect of water deficit on crop production. Yield per vine, clusters per vine, and cluster weight of fully irrigated (C, controls) and deficit irrigated (D, water deficit) grapevines reported as the average ± the standard error. Numbers in bold indicate significant differences between treatments (P < 0.05) identified by one-way ANOVA (n = 4). (DOC 29 kb) [file 12870_2016_760_MOESM2_ESM.doc]

| **Table S2.** Effect of water deficit on crop production. Yield per vine, clusters per vine, and cluster weight of fully irrigated (C, controls) and deficit irrigated (D, water deficit) grapevines reported as the average ± the standard error. Numbers in bold indicate significant differences between treatments (*P*<0.05) identified by one-way ANOVA (n=4). | | | | | | |
| --- | --- | --- | --- | --- | --- | --- |
| **Year** | **Yield per Vine (kg)** | | **Clusters per vine** | | **Cluster Weight (g)** | |
|  | C | D | C | D | C | D |
| 2012 | **3.27±0.2** | **2.52±0.3** | 20.1±2.6 | 18.4±2.0 | **170.5±10.8** | **139.4±15.5** |
