# Supplementary material for: Transcriptome and metabolite profiling reveals that prolonged drought modulates the phenylpropanoid and terpenoid pathway in white grapes (Vitis vinifera L.)
Source: BMC Plant Biol. 2016 Mar 21;16:67. doi: 10.1186/s12870-016-0760-1 (PMC4802899; doi:10.1186/s12870-016-0760-1)
Supplement: Additional file 3: Table S3. — List of phenolics, carotenoids, tocopherols and free VOCs identified in the study. (DOC 88 kb) [file 12870_2016_760_MOESM3_ESM.doc]

| **Table S3.** List of phenolics, carotenoids, and free VOCs identified in this study using UHPLC-MS/MS, HPLC-DAD, and HS-SPME-GC-MS platforms. | | |
| --- | --- | --- |
| **Compound** | **Chemical Class** | **Analytical Platform** |
| Ellagic acid | Benzoic and Cinnamic acids | UHPLC-MS/MS |
| Gallic acid | Benzoic and Cinnamic acids | UHPLC-MS/MS |
| Methyl gallate | Benzoic and Cinnamic acids | UHPLC-MS/MS |
| *trans*-Caftaric acid | Benzoic and Cinnamic acids | UHPLC-MS/MS |
| *trans*-Coutaric acid | Benzoic and Cinnamic acids | UHPLC-MS/MS |
| *trans*-Fertaric acid | Benzoic and Cinnamic acids | UHPLC-MS/MS |
| Astringin | Stilbenoids | UHPLC-MS/MS |
| Pallidol | Stilbenoids | UHPLC-MS/MS |
| *cis*-Piceid | Stilbenoids | UHPLC-MS/MS |
| *trans*-Piceid | Stilbenoids | UHPLC-MS/MS |
| *trans*-ε-Viniferin | Stilbenoids | UHPLC-MS/MS |
| Phlorizin | Hydrochalcones | UHPLC-MS/MS |
| Trilobatin | Hydrochalcones | UHPLC-MS/MS |
| Kaempferol-3-*O*-glucoside | Flavonols | UHPLC-MS/MS |
| Quercetin-3-*O*-glucoside | Flavonols | UHPLC-MS/MS |
| Quercetin-3-*O*-glucoronide | Flavonols | UHPLC-MS/MS |
| Rutin | Flavonols | UHPLC-MS/MS |
| *(+)-*Catechin | Flavan-3-ols | UHPLC-MS/MS |
| *(-)-*Epicatechin | Flavan-3-ols | UHPLC-MS/MS |
| *(-)*-Epicatechin gallate | Flavan-3-ols | UHPLC-MS/MS |
| *(-)*-Epigallocatechin | Flavan-3-ols | UHPLC-MS/MS |
| *(-)*-Epigallocatechin gallate | Flavan-3-ols | UHPLC-MS/MS |
| *(+)*-Gallocatechin | Flavan-3-ols | UHPLC-MS/MS |
| Procyanidin B1 | Proanthocyanidins | UHPLC-MS/MS |
| Procyanidin B2+B4 | Proanthocyanidins | UHPLC-MS/MS |
| Procyanidin B3 | Proanthocyanidins | UHPLC-MS/MS |
| Caffeic acid + catechin condensation product | Others | UHPLC-MS/MS |
| Antheraxanthin | Carotenoids | HPLC-DAD |
| *β*-Carotene | Carotenoids | HPLC-DAD |
| *9-(Z)-β*-Carotene | Carotenoids | HPLC-DAD |
| Lutein | Carotenoids | HPLC-DAD |
| Lutein, 5-6 epoxide | Carotenoids | HPLC-DAD |
| *9-(Z)*-Neoxanthin | Carotenoids | HPLC-DAD |
| Violaxanthin | Carotenoids | HPLC-DAD |
| Zeaxanthin | Carotenoids | HPLC-DAD |
| *α*-Tocopherol | Tocopherols | HPLC-DAD |
| *γ*-Tocopherol | Tocopherols | HPLC-DAD |
| Hexanoic acid | Acids | HS-SPME-GC-MS |
| Heptanol | Alcohols | HS-SPME-GC-MS |
| Hexanol | Alcohols | HS-SPME-GC-MS |
| 2-Hexenol | Alcohols | HS-SPME-GC-MS |
| 3-Hexenol | Alcohols | HS-SPME-GC-MS |
| Nonanol | Alcohols | HS-SPME-GC-MS |
| Octanol | Alcohols | HS-SPME-GC-MS |
| 1-Octen-3-ol | Alcohols | HS-SPME-GC-MS |
| Benzaldehyde | Aldehydes | HS-SPME-GC-MS |
| *(E,E)*-2,4-Heptadienal | Aldehydes | HS-SPME-GC-MS |
| Heptanal | Aldehydes | HS-SPME-GC-MS |
| *(E)*-2-Heptenal | Aldehydes | HS-SPME-GC-MS |
| *(E,E)*-2,4-Hexadienal | Aldehydes | HS-SPME-GC-MS |
| Hexanal | Aldehydes | HS-SPME-GC-MS |
| *(E)*-2-Hexenal | Aldehydes | HS-SPME-GC-MS |
| Nonanal | Aldehydes | HS-SPME-GC-MS |
| *(E)*-2-Nonenal | Aldehydes | HS-SPME-GC-MS |
| *(E)*-2-Octenal | Aldehydes | HS-SPME-GC-MS |
| *(E)*-2-Pentenal | Aldehydes | HS-SPME-GC-MS |
| *β*-Cyclocitral | C10-Norisoprenoids | HS-SPME-GC-MS |
| *β*-Damascenone | C13-Norisoprenoids | HS-SPME-GC-MS |
| *β*-Ionone | C13-Norisoprenoids | HS-SPME-GC-MS |
| *β*-Ionone-5-6-epoxide | C13-Norisoprenoids | HS-SPME-GC-MS |
| 3-Hexenyl acetate | Esters | HS-SPME-GC-MS |
| 6-Methyl-5-hepten-2-one | Ketones | HS-SPME-GC-MS |
| 1-Octen-3-one | Ketones | HS-SPME-GC-MS |
| *trans*-Caryophyllene | Terpenes | HS-SPME-GC-MS |
| Citronellol | Terpenes | HS-SPME-GC-MS |
| Geraniol | Terpenes | HS-SPME-GC-MS |
| Geranyl acetone | Terpenes | HS-SPME-GC-MS |
| Hotrienol | Terpenes | HS-SPME-GC-MS |
| *α*-Humulene | Terpenes | HS-SPME-GC-MS |
| Linalool | Terpenes | HS-SPME-GC-MS |
| Linalool oxide A | Terpenes | HS-SPME-GC-MS |
| Linalool oxide B | Terpenes | HS-SPME-GC-MS |
| Nerol | Terpenes | HS-SPME-GC-MS |
| *α*-Terpineol | Terpenes | HS-SPME-GC-MS |
|  |  |  |
|  |  |  |
