# Supplementary figures and images for: Transcriptome and metabolite profiling reveals that prolonged drought modulates the phenylpropanoid and terpenoid pathway in white grapes (Vitis vinifera L.)
Source: BMC Plant Biol. 2016 Mar 21;16:67. doi: 10.1186/s12870-016-0760-1 (PMC4802899; doi:10.1186/s12870-016-0760-1)

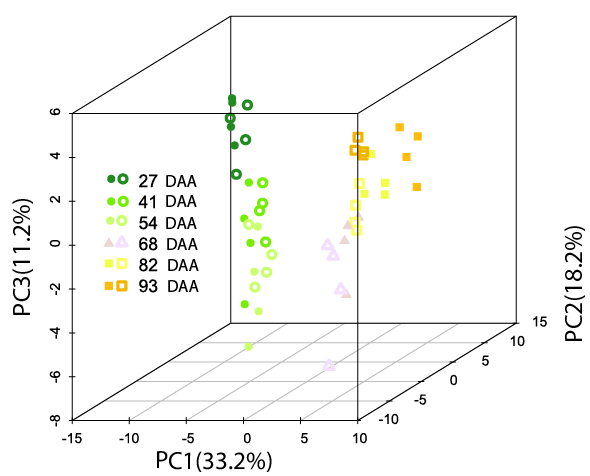

Supplement: Additional file 4: Figure S1. — Principal component analysis (PCA) of the berry secondary metabolite profile of 48 independent samples collected from C and D vines at 27, 41, 54, 68, 82, and 93 DAA. Full and open symbols identify C and D berries, respectively. (PNG 27 kb) [file 12870_2016_760_MOESM4_ESM.png]

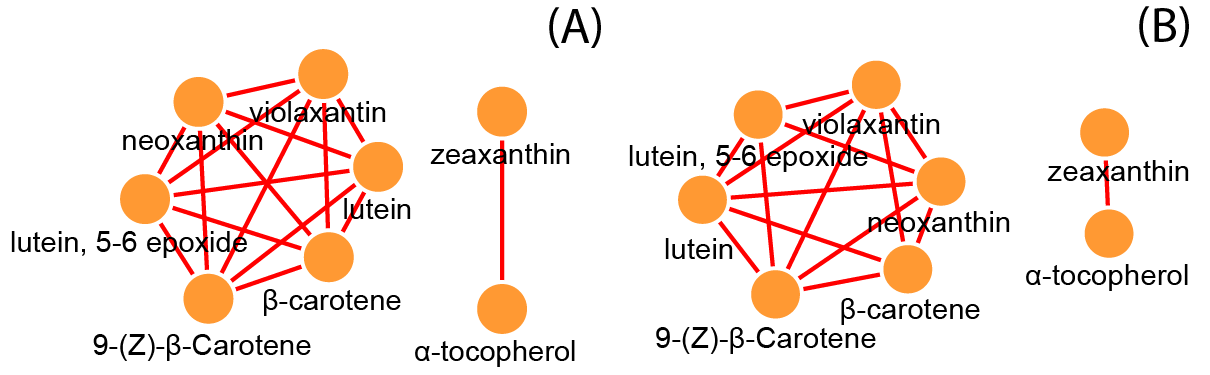

Supplement: Additional file 9: Figure S5. — Carotenoid network. Network representation of carotenoids in C (A) and D (B) berries during development. Nodes represent ‘metabolites’ and edges represent ‘relationships’ between any two metabolites. Edges colored in red and blue represent significant (P < 0.001) positive and negative correlations, respectively. Metabolites in bold indicate a significant effect (P < 0.05) of water deficit on the concentration of that metabolite at one or more developmental stages. Number of correlating edges, average node neighborhood, and clustering coefficient were similar between C and D networks. (PNG 63 kb) [file 12870_2016_760_MOESM9_ESM.png]

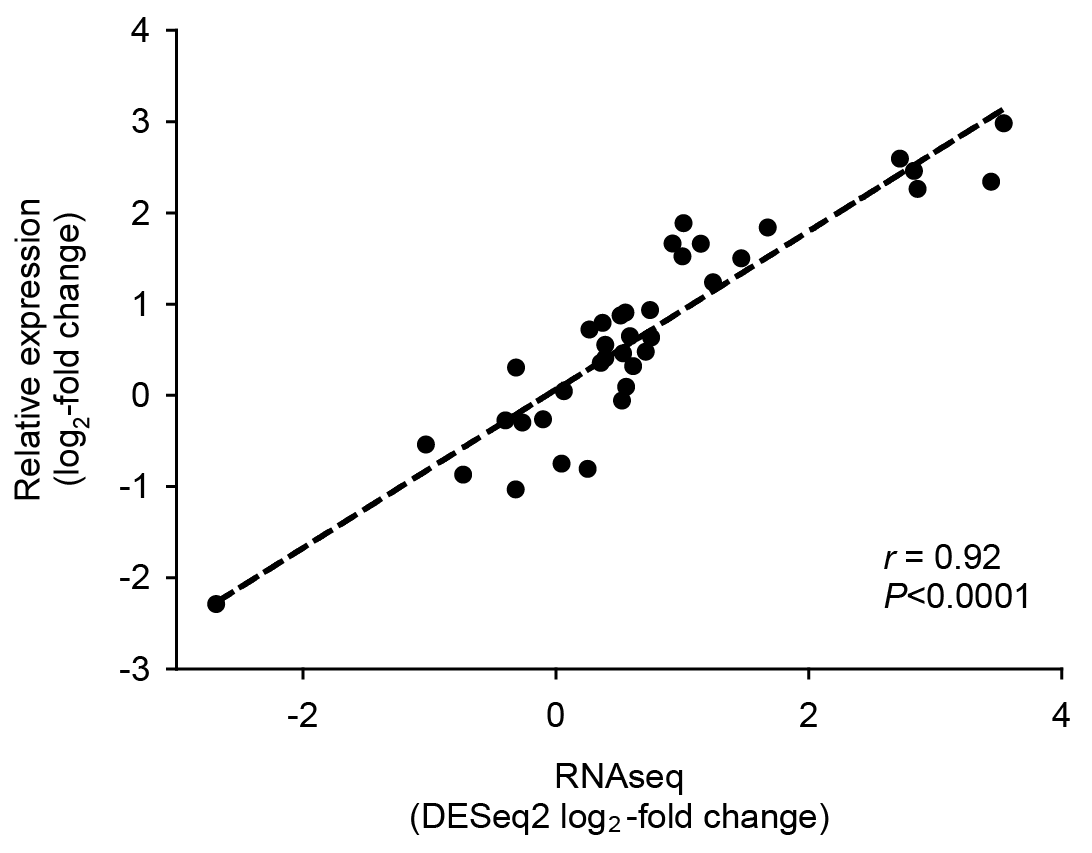

Supplement: Additional file 12: Figure S6. — Scatterplot of the correlation between the fold changes (log2(D/C)) in the expression levels of the 15 genes reported in Additional file 11: Table S5 obtained by RNA-seq and qPCR analyses. (TIF 1009 kb) [file 12870_2016_760_MOESM12_ESM.tif]

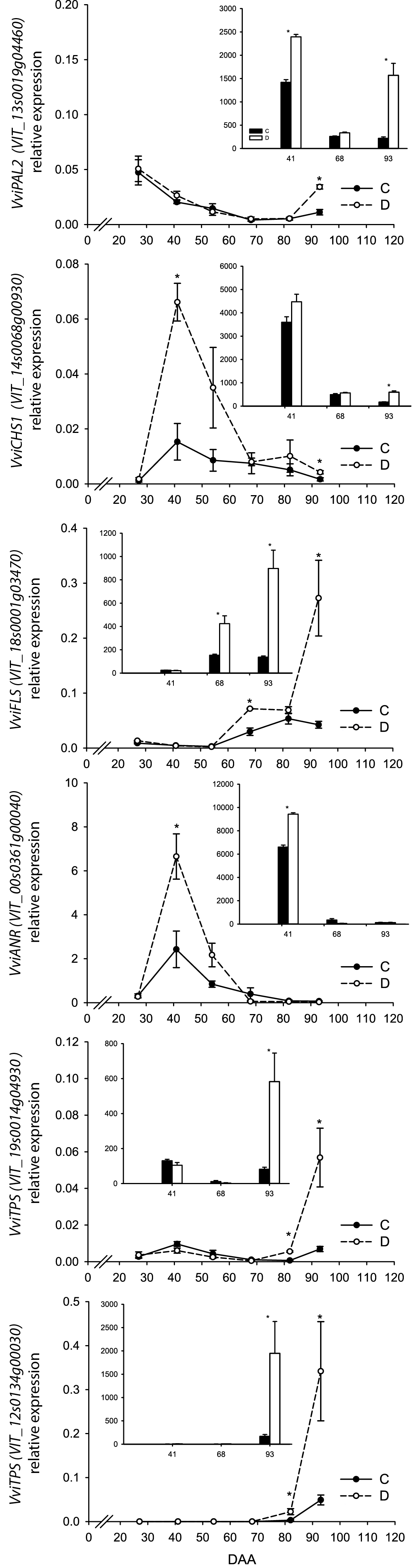

Supplement: Additional file 14: Figure S7. — Target gene expression analysis. Impact of water deficit on transcript abundance of selected genes of the phenylpropanoid, flavonoid, and terpenoid pathway. Using qPCR, gene expression was analyzed at each sampling time. Gene expression levels analyzed with RNA-sequencing at 41, 68, and 93 DAA are reported in inset graphs for comparison. Bars represent ± SE. Asterisks indicate significant differences between treatments at P < 0.05 (*). (PNG 275 kb) [file 12870_2016_760_MOESM14_ESM.png]

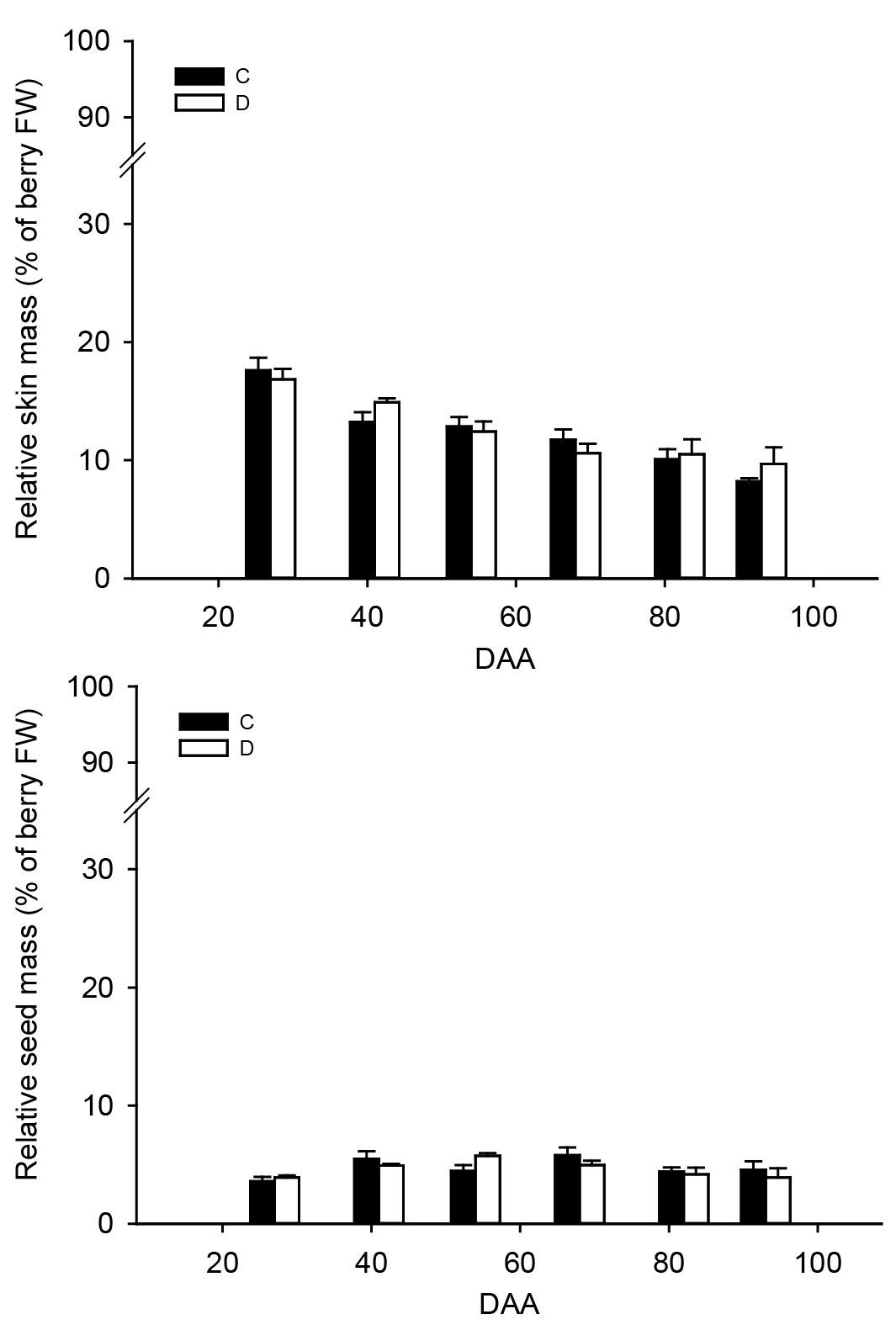

Supplement: Additional file 15: Figure S8. — Evolution of the relative skin and seed masses expressed as % of berry fresh weight (FW) across development in C and D berries. (TIF 1797 kb) [file 12870_2016_760_MOESM15_ESM.tif]
