# Supplementary material for: Transcriptome and metabolite profiling reveals that prolonged drought modulates the phenylpropanoid and terpenoid pathway in white grapes (Vitis vinifera L.)
Source: BMC Plant Biol. 2016 Mar 21;16:67. doi: 10.1186/s12870-016-0760-1 (PMC4802899; doi:10.1186/s12870-016-0760-1)
Supplement: Additional file 5: Figure S2. — Phenolic profiling. Trends of phenolic concentrations in C and D berries during fruit development. (DOCX 358 kb) [file 12870_2016_760_MOESM5_ESM.docx]

**Figure S2**

Benzoic and cinnamic acids

Stilbenoids

Hydroxychalcones

Flavonols

Flavan-3-ols

Proanthocyanidins

Others
