# Supplementary material for: Transcriptome and metabolite profiling reveals that prolonged drought modulates the phenylpropanoid and terpenoid pathway in white grapes (Vitis vinifera L.)
Source: BMC Plant Biol. 2016 Mar 21;16:67. doi: 10.1186/s12870-016-0760-1 (PMC4802899; doi:10.1186/s12870-016-0760-1)
Supplement: Additional file 6: Figure S3. — Carotenoid and tocopherol profiling. Trends of carotenoid and tocopherol concentrations in C and D berries during fruit development. (DOCX 163 kb) [file 12870_2016_760_MOESM6_ESM.docx]

**Figure S3**

Carotenoids

Tocopherols
