# Supplementary material for: Transcriptome and metabolite profiling reveals that prolonged drought modulates the phenylpropanoid and terpenoid pathway in white grapes (Vitis vinifera L.)
Source: BMC Plant Biol. 2016 Mar 21;16:67. doi: 10.1186/s12870-016-0760-1 (PMC4802899; doi:10.1186/s12870-016-0760-1)
Supplement: Additional file 10: Table S4. — RNA sequencing analysis metrics. Transcriptome analyses were performed in C and D berries at three selected berry developmental stages (41 DAA, before ripening; 68 DAA, beginning of ripening; 93 DAA, late ripening) using an Illumina HiSeq platform. (DOC 54 kb) [file 12870_2016_760_MOESM10_ESM.doc]

| **Table S4.** RNA sequencing analysis metrics. Transcriptome analyses were performed in C and D berries at three selected berry developmental stages (41 DAA, before ripening; 68 DAA, beginning of ripening; 93 DAA, late ripening) using an Illumina HiSeq platform. | | | | | | | |
| --- | --- | --- | --- | --- | --- | --- | --- |
| **Sampling**  **(DAA)** | **Treatment** | **Biol. Rep.** | **Sequenced**  **Reads** | **Trimmed**  **and Filtered Reads** | **Mapped**  **Reads** | **Unique**  **Reads** | **Expressed**  **Genes** |
| 41 | C | 1 | 28,142,936 | 27,754,632 | 25,988,030 | 24,755,747 | 23,610 |
| 41 | C | 2 | 30,305,610 | 29,876,940 | 27,973,973 | 26,562,085 | 23,599 |
| 41 | C | 3 | 28,671,516 | 28,222,582 | 26,091,160 | 24,772,968 | 23,540 |
| 41 | D | 1 | 28,149,289 | 27,778,718 | 26,187,110 | 24,902,068 | 23,514 |
| 41 | D | 2 | 33,830,303 | 33,405,718 | 31,568,524 | 30,062,254 | 23,674 |
| 41 | D | 3 | 32,599,088 | 32,104,203 | 30,020,648 | 28,489,098 | 23,680 |
| 68 | C | 1 | 28,782,803 | 28,323,977 | 26,348,993 | 25,223,216 | 22,237 |
| 68 | C | 2 | 25,079,582 | 24,669,614 | 22,976,645 | 21,995,680 | 22,390 |
| 68 | C | 3 | 28,125,993 | 27,686,829 | 25,741,134 | 24,643,657 | 22,791 |
| 68 | D | 1 | 26,901,387 | 26,446,179 | 24,543,406 | 23,239,648 | 21,956 |
| 68 | D | 2 | 30,338,107 | 29,825,731 | 27,814,720 | 26,486,281 | 22,100 |
| 68 | D | 3 | 28,142,082 | 27,663,249 | 25,648,895 | 24,384,788 | 22,077 |
| 93 | C | 1 | 23,482,080 | 23,170,055 | 21,908,218 | 21,010,267 | 21,799 |
| 93 | C | 2 | 28,620,256 | 28,210,433 | 26,438,882 | 25,361,424 | 22,073 |
| 93 | C | 3 | 27,093,095 | 26,687,888 | 24,966,036 | 23,949,887 | 22,254 |
| 93 | D | 1 | 32,448,861 | 32,030,568 | 30,407,000 | 29,224,179 | 22,774 |
| 93 | D | 2 | 32,234,615 | 31,726,941 | 29,721,915 | 28,544,263 | 22,783 |
| 93 | D | 3 | 27,177,622 | 26,846,929 | 25,470,062 | 24,417,385 | 22,408 |
