# Supplementary material for: Transcriptome and metabolite profiling reveals that prolonged drought modulates the phenylpropanoid and terpenoid pathway in white grapes (Vitis vinifera L.)
Source: BMC Plant Biol. 2016 Mar 21;16:67. doi: 10.1186/s12870-016-0760-1 (PMC4802899; doi:10.1186/s12870-016-0760-1)
Supplement: Additional file 11: Table S5. — List of genes assayed for expression by qPCR. For each, the coefficient of correlation between RNA-Seq data and qPCR data, the P value, forward and reverse primers used, and literature references are shown. (DOC 38 kb) [file 12870_2016_760_MOESM11_ESM.doc]

| **Table S5.** List of genes assayed for expression by qPCR. For each, the coefficient of correlation between RNA-Seq data and qPCR data, *P* value, forward and reverse primers used, and literature references are shown. | | | | | |
| --- | --- | --- | --- | --- | --- |
| **Gene ID** | **R ^2^ Correlation Coefficient** | ***P* value** | **Forward / Reverse primer sequence** | | **Reference** |
| *VviPAL2 (VIT_13s0019g04460)* | 0.993 | 3.02  e-04 | atgaggtgaagcggatggtg / gcctttactccctctctcgc | Newly designed | |
| *VviCHS1 (VIT_14s0068g00930)* | 0.893 | 6.57  e-03 | agccagtgaagcaggtagcc / gtgatccggaagtagtaat | Goto-Yamamoto et al. 2002, Plant Science | |
| *VviCHS2 (VIT_14s0068g00920)* | 0.999 | 5.63  e-06 | tctgagcgagtatgggaaca / agggtagctgcgtaggttgg | Goto-Yamamoto et al. 2002, Plant Science | |
| *VviCHS3 (VIT_05s0136g00260)* | 0.998 | 8.18  e-06 | gtttcggaccagggctcact / ggcaagtaaagtggaaacag | Goto-Yamamoto et al. 2002, Plant Science | |
| *VviF3H_2 (VIT_18s0001g14310)* | 0.792 | 0.017 | ctgtggtgaactccgactgc / caaatgttatgggctcctcc | Jeong et al. 2004, Plant Science | |
| *VviLDOX (VIT_02s0025g04720)* | 0.967 | 9.23  e-04 | agggaagggaaaacaagtag / actctttggggattgactgg | Jeong et al. 2004, Plant Science | |
| *VviLAR1 (VIT_01s0011g02960)* | 0.960 | 5.48  e-04 | caggaggctatggagaagatac / acgcttctctctgtacatgttg | Bogs et al. 2005, Plant Physiology | |
| *VviANR (VIT_00s0361g00040)* | 0.981 | 9.92  e-05 | agcaggttgcgactttgtct / accagacctgtcccatcaag | Castellarin et al. 2007, Plant Cell and Environment | |
| *VviMybPA1 (VIT_15s0046g00170)* | 0.962 | 2.61  e-04 | ttgacggggttgacttcttc / gagtagtgattcggcgaagg | Terrier et al. 2009, Plant Physiology | |
| *VviFLS (VIT_18s0001g03470)* | 0.993 | 7.25  e-05 | tggggttaggtctgggagag / aacctgcaagccctgaactt | Newly designed | |
| *VviTPS_15 (VIT_18s0001g05290)* | 0.881 | 0.010 | ggaatgcctcaaacctactgc / ggttaatgaagaaagccgcaatg | Newly designed | |
| *VviTPS_28 (VIT_19s0014g04930)* | 0.991 | 5.59  e-05 | cgggtggtggaatgctactt / acctctcaactgcttcggtg | Newly designed | |
| *VviTPS_35 (VIT_12s0134g00030)* | 0.998 | 6.02  e-05 | ctctgaggaaagtgctcgtga / ccttgatctagcgtccggag | Newly designed | |
| *VviNCED3 (VIT_19s0093g00550)* | 0.997 | 4.22  e-06 | tttgtgcacgacgagaagac / agggaactcgtgagggaagt | Newly designed | |
| *VviCCD4b (VIT_02s0087g00930)* | 0.968 | 5.19  e-04 | atctgaaaacggggacagtg / acgtccagcttcacaattcc | Newly designed | |
